# Development and evaluation of [^11^C]DPA-813 and [^18^F]DPA-814: novel TSPO PET tracers insensitive to human single nucleotide polymorphism *rs6971*

**DOI:** 10.1007/s00259-025-07109-1

**Published:** 2025-02-05

**Authors:** Wissam Beaino, Esther JM Kooijman, Eryn L. Werry, Rens J. Vellinga, Johan Van den Hoek, Greta Sohler, Grace A. Cumbers, Elijah Genetzakis, Edward D. Harvey-Latham, Robert C. Schuit, Michael Kassiou, Albert D. Windhorst, Jonathan J. Danon

**Affiliations:** 1 Department Radiology & Nuclear Medicine, Amsterdam UMC Location Vrije Universiteit Amsterdam, De Boelelaan 1117, Amsterdam, 1081 HV The Netherlands; 2 Amsterdam Neuroscience, Brain Imaging, Amsterdam, The Netherlands; 3 School of Chemistry, Faculty of Science, University of Sydney, Sydney, NSW 2050 Australia; 4 Central Clinical School, Faculty of Medicine and Health, University of Sydney, Sydney, NSW 2050 Australia

**Keywords:** Translocator protein 18 kDa, Positron emission tomography, Neuroinflammation, Polymorphism, Experimental autoimmune encephalomyelitis, Autoradiography

## Abstract

**Purpose:**

The translocator protein 18 kDa (TSPO) is a widely used marker for imaging neuroinflammation via Positron Emission Tomography (PET). However, the vast majority of reported TSPO PET tracers display low binding affinity to a common isoform of human TSPO (*rs6971*; A147T), making them unsuitable for universal use in the general population. In this study, we have developed and preclinically validated two novel tracers designed to image TSPO in patients of all genotypes.

**Methods:**

Novel analogues of known TSPO ligands were synthesised, evaluated for TSPO binding affinity in vitro (membranes prepared from transfected HEK-293T cells expressing wild-type (WT) or A147T TSPO) and radiolabelled with carbon-11 or fluorine-18. They were evaluated in situ (autoradiography on genotyped human brain tissue) and in vivo (rat, both WT and clinically relevant experimental autoimmune encephalomyelitis (EAE) neuroinflammation model) as potential polymorphism-insensitive TSPO PET tracers.

**Results:**

Two new TSPO ligands, DPA-813 and DPA-814, displayed equivalent single-digit nanomolar binding affinities in vitro towards both human TSPO isoforms. [^11^C]DPA-813 and [^18^F]DPA-814 were synthesised in moderate radiochemical yields, high radiochemical purity, and high molar activity. Autoradiography on human MS tissues showed high specific binding for both tracers, irrespective of the TSPO isoform. The tracers demonstrated high plasma stability after 45 min and no brain metabolism with > 99% intact tracer. Biodistribution in WT animals indicated good brain uptake for both tracers (0.28 and 0.41%ID/g for [^18^F]DPA-814 and [^11^C]DPA-813, respectively). PET imaging in the clinically relevant EAE neuroinflammation model in rats showed significantly higher uptake of [^11^C]DPA-813 and [^18^F]DPA-814 in the spinal cord of the EAE rats compared to the controls.

**Conclusion:**

We have developed two novel PET tracers that display indiscriminately high binding affinity to both common isoforms of human TSPO, show favourable metabolic stability and brain penetration in rats, and significantly higher uptake in the spinal cord of a neuroinflammatory rat model of multiple sclerosis. Going forward, first-in-human clinical validation will mark a critical juncture in the development of these tracers, which could offer substantial improvements over existing imaging tools for detecting neuroinflammation, irrespective of genetic variations.

**Supplementary Information:**

The online version contains supplementary material available at 10.1007/s00259-025-07109-1.

## Introduction

The translocator protein 18 kDa (TSPO), found predominantly in steroidogenic tissue, is a mitochondrial membrane protein with numerous purported biological roles in health and disease [1]. Notably, TSPO has been used as a biomarker of neuroinflammation due to its increased expression during inflammatory processes, particularly in activated microglia [2, 3] but also in astrocytes [4, 5] and neurons [6]. As a result, imaging the expression and distribution of TSPO via positron emission tomography (PET) has been demonstrated to reflect the inflammatory component of various central nervous system (CNS) disorders [7]. Despite prevailing questions surrounding the functions and cellular sources of TSPO [8], its expression discrepancies between rodents and humans [9], and its role in other non-inflammatory physiological processes [10], TSPO PET remains the primary method for assessing the inflammatory component of CNS disease pathology in both clinical and pre-clinical settings [7].

Currently used TSPO tracers suffer from critical limitations. (*R*)-[^11^C]PK 11195 is still widely used despite exhibiting low brain uptake, high plasma protein binding, and poor signal-to-noise ratios [11–13]. Second-generation TSPO ligands have resolved these issues, with many reported to display superior specific binding and pre-clinical imaging profiles to (*R*)-[^11^C]PK 11195. Notable examples are [^11^C]PBR28 [14], [^11^C]DPA-713 [15], [^18^F]DPA-714 [16], and [^18^F]FEPPA [17]., However, a single-nucleotide polymorphism (*rs6971*) in humans results in a non-conservative substitution of alanine for threonine at residue 147 (A147T) of TSPO [18, 19]. This mutation significantly reduces the binding affinities of second-generation ligands and leads to the presence of high-, mixed-, and low-affinity binders (HABs, MABs, and LABs) [20]. The reduction in affinity leads to variation in PET signal for human subjects, who must be genotyped prior to imaging to determine their binding status. Exclusion of LABs, and in many cases MABs, from clinical studies due to this mutation remains an enduring limitation of TSPO PET that has hindered its uptake into clinical practice.

Recent years have seen reports of ‘third-generation’ TSPO tracers that claim to bind indiscriminately to wild-type (WT) and A147T TSPO. Of these, [^18^F]GE180 displays moderate in vitro binding affinity and a 5-fold reduction in affinity for A147T TSPO in vitro [21], as well as poor brain uptake and high activity in blood vessels [22–24]. Related tracer [^18^F]GE387 displayed slightly improved affinity at A147T TSPO but 3-fold reduced affinity to WT TSPO versus [^18^F]GE180 [21]. [^11^C]ER176 still exhibits undesirable isoform discrimination *in vivo.* [25] Another candidate, [^18^F]BS224, has shown insensitivity to the polymorphism in vitro as well as encouraging imaging potential in LPS-induced rat models of neuroinflammation but has yet to be translated to higher-order organisms or evaluated against human tissue [26]. [^18^F]LW223 has been used to map macrophage-driven inflammation after myocardial infarction independent of the *rs6971* polymorphism [27]. Initial studies on stability and biodistribution in mice [28], non-human primates, and humans [29] have also shown promise. However, these challengers still require further clinical validation.

The current work describes the in vitro and in vivo pre-clinical evaluation of two novel TSPO tracer candidates insensitive to the *rs6971* polymorphism: [^11^C]DPA-813 and [^18^F]DPA-814. These tracers were (i) confirmed as high affinity, non-discriminating TSPO binders in HEK cells transfected with WT or A147T TSPO and human brain tissue from genotyped multiple sclerosis patients, (ii) characterised for biodistribution and metabolic stability in WT rats, and (iii) evaluated for the ability to image neuroinflammation in an EAE rat model of multiple sclerosis. Our findings pave the way for clinical translation of these tracers, adding to the growing number of potential TSPO PET tracers that may be used in patients of all genotypes.

## Materials and methods

### Chemistry

Synthetic procedures for DPA-813, DPA-814 and their respective radiolabelling precursors were adapted from published methods and are included in Supplementary Information. Radiolabelling precursors and non-radioactive reference standards of [^11^C]DPA-713 and [^18^F]DPA-714 were synthesised as per published protocols [30, 31]. 

### In vitro binding

Binding affinities (K_i_) to wild-type (WT) and A147T human TSPO were assessed using a previously published method [32]. HEK-293T cells were stably transfected with WT and A147T TSPO. Membranes were isolated from each cell line using an Ultra-Turrax hand-held homogeniser. These cells have been previously validated as an in vitro model for low- and high-affinity TSPO binding. This involves diluting WT (20 µg/well) and A147T (5 µg/well) membranes in 50 mM trisaminomethane (Tris) HCl (pH 7.4) and incubating them at 4 °C for 90 min with ~ K_d_ concentration of [^3^H]PK 11195 (10 nM; PerkinElmer) and test compounds (0.3 nM to 10 µM). Non-specific binding was determined using a saturating concentration (1 µM) of non-tritiated PK 11195. This non-specific binding contributed less than 10% of total binding. Reactions were halted by filtering through a 96-well glass-fibre filter plate (Millipore), followed by eight washes with ice-cold 50 mM Tris HCl. Microscint 0 was added to the plate and resultant radioactivity-related photons were collected by a Microbeta 2450 (PerkinElmer). K_i_ values were fit to a competition binding curve fit on non-specific binding-corrected data using GraphPad Prism 10.0 (GraphPad). Results are presented as mean ± standard error of the mean (SEM) from at least three independent experiments. Binding affinities (K_i_) to rat TSPO were measured in duplicate by Eurofins Panlabs Discovery Services (assay #226700) (Supplementary Fig. 12). Single-dose (10 µM) off-target in vitro screening was performed in duplicate by Eurofins Panlabs Discovery Services (Supplementary Table 5).

### Lipophilicity measurements

Partition coefficient values (logD) were estimated at pH 7.4 by correlating chromatographic retention properties against the characteristics of a series of standard compounds with known partition coefficient values. The method employed a gradient HPLC based on a previously published method [33].

### Predicted CNS permeability calculations

CNS PET Multiparameter Optimization (MPO) scores were calculated using MarvinSketch v23.13 and the method outlined in reference [34]. Scores ≥ 3 indicate a high likelihood of passive CNS permeability.

### Radiolabelling

#### Synthesis of [^11^C]DPA-813

[^11^C]CO_2_ produced by a cyclotron (Cyclone 18/9, IBA, Louvain-la-Neuve, Belgium) was trapped in a stainless steel coil cooled with liquid nitrogen. The trapped [^11^C]CO_2_ was then transferred to the hot cell with a helium gas flow of 10 mL/min, directed into a reaction vessel through a cartridge filled with Sicapent. This vessel contained 100 µL of a 0.1 M LiAlH_4_ solution in THF (ABX, Radeberg, Germany). Once all the [^11^C]CO_2_ was collected, the THF was evaporated by heating to 130 °C while blowing helium at 20 mL/min. When 130 °C was reached, 200 µL of HI (57%, Merck Millipore, Billerica, USA) was added, producing [^11^C]methyl iodide. This product was distilled from the reaction mixture with a 20 mL/min helium flow into a second reaction vessel containing the precursor **1** (1.0 mg, 2.6 mmol) dissolved in 300 µL of acetonitrile and 2.5 µL of a 0.5 M NaOH solution in water. This vessel also contained a cartridge filled with 50% NaOH pellets and 50% Sicapent. The mixture was heated for 5 min at 100 °C, then cooled to 20 °C and injected into a semi-preparative HPLC system (Reprospher C18-DE 5µ 50 × 8 mm column with a 40/60 acetonitrile/water + 0.1% diisopropylethylamine eluent at 3 mL/min) for purification. [^11^C]DPA-813 eluted at 12 min and was collected into 60 mL of water. This solution was passed over a Sep-Pak tC18 cartridge (Waters, Etten-Leur, The Netherlands) and washed with 20 mL of sterile water (B. Braun, Sempach, Switzerland). The product was then eluted with 1.0 mL of sterile ethanol (96%) (Clinical Pharmacology and Pharmacy; Albert Schweitzer Hospital, Dordrecht), diluted with 10 mL of 0.9% sodium chloride solution (B. Braun, Sempach, Switzerland), and filtered through a sterile Millex GV 0.22 μm filter (Merck Millipore, Billerica, USA). The radiochemical purity of [^11^C]DPA-813, assessed by HPLC (Merck Chromolith Performance RP18e column; 4.6 × 100 mm, water/acetonitrile 55/45 eluent at 1 mL/min), was over 98% with no UV impurities detected. The molar activity of [^11^C]DPA-813 exceeded 70 GBq/µmol at the end of synthesis, with a decay-corrected radiochemical yield of 15–25% based on [^11^C]CO_2_.

#### Synthesis of [^18^F]DPA-814

The [^18^F]fluoride produced by the Cyclone 18/9 cyclotron was captured on a Machery Nagel PS-HCO3 anion exchange column (ABX, Radeberg, Germany). It was eluted with 0.5 mL of a 0.1 M potassium sulfate solution into a pre-cleaned vial containing N, N-bistriflyl aniline dissolved in 1 mL of DMF. This mixture was heated to 40 °C to generate [^18^F]triflylfluoride, which was then bubbled out, dried over a Sicapent column, and trapped in a screw cap reaction vessel containing 0.25 mL of acetonitrile with 3.75 mg (10.0 µmol) of Kryptofix 2.2.2 (Merck Millipore, Billerica, USA) and 0.75 mg (7.5 µmol) of potassium hydrogen carbonate (Aldrich, Zwijndrecht, The Netherlands). The solution was evaporated to dryness under a helium flow (50 mL/min) and reduced pressure (10–15 hPa) at 100 °C. The precursor **2** (2.0 mg, 4.6 µmol), dissolved in 250 µL of dry DMSO, was then added to the reaction vessel. This mixture was heated for 7 min at 120 °C, cooled to room temperature, and quenched with 3.0 mL of water. The resulting solution underwent HPLC purification using an XTerra RP18 5 μm, 250 × 10 mm column with a 0.1 M ammonium acetate/acetonitrile (50/50) eluent at a flow rate of 4 mL/min. The product eluted at approximately 10 min and was collected in 50 mL of water. This solution was passed through a Sep-Pak tC18 (Waters) and washed with 20 mL of sterile water for injection (B. Braun). The product was eluted from the Sep-Pak with 1.5 mL of sterile ethanol (96%, Clinical Pharmacology and Pharmacy) and diluted with 15 mL of sterile 0.9% sodium chloride solution (B. Braun, Sempach, Switzerland). The final solution was filtered through a sterile Millex GV 0.22 μm filter (Merck Millipore, Billerica, USA). The radiochemical purity was determined to be greater than 95% using HPLC on an Acquity HSS T3 75 × 2.1 mm, 1.8 μm UPLC column with a water/acetonitrile/TFA (58/42/0.1) eluent at a flow rate of 0.5 mL/min. The molar activity exceeded 28 GBq/µmol, although some UV impurities amounting to about 5% of the DPA-814 surface area were observed. The decay-corrected radiochemical yield from [^18^F]fluoride was calculated to be 30–40%.

### Animals and EAE model

Wild-type 10–12 weeks-old female Wistar/*RccHan* (WIST) rats (Inotiv, the Netherlands) were used for biodistribution and metabolite studies. Experimental autoimmune encephalomyelitis (EAE) was induced in 10–15 weeks-old female Lewis/*HanHsd* rats (Inotiv, the Netherlands). EAE was induced as previously described [35]. Briefly, rats were immunised s.c. with an emulsion (200 µL) containing 200 µg synthetic guinea pig myelin basic protein peptide (gpMBP 69–88), 500 µg Mycobacterium tuberculosis type 37HRa (Difco, Detroit, MI, USA), 100 µL complete Freund’s adjuvant (CFA) (Difco), and 100 µL sterile water. Pertussis toxin (400 ng) (Gibco) was injected i.p. at day 0 and day 2 post-immunisation. Rats were examined daily, and clinical scores were graded on a scale from 1 to 5 for neurological signs. Animals were housed under standard laboratory conditions with water and food ad libitum. Animals immunised only with CFA (200 µL) supplemented with Mycobacterium tuberculosis type 37HRa (6 mg/mL) were used as a control. Animal experiments were performed following the European Community Council Directive (2010/63/EU) for laboratory animal care and the Dutch Law on animal experimentation. The experimental protocol was validated and approved by the Central Committee for Animal Experimentation (CCD) and the local committee on animal experimentation of the Amsterdam University Medical Centers.

### Metabolites analysis

Metabolite analysis was conducted following the method outlined in a previous study [35]. Wistar/RccHan (WIST) rats (Inotiv, the Netherlands) received intravenous injections of either [^11^C]DPA-813 (30–50 MBq) or [^18^F]DPA-814 (20–30 MBq). Rats were euthanized at 5, 15, and 45 min post-injection (*n* = 4 per time point). Plasma was separated from blood then processed through a C18 Sep-Pak (Waters, Milford, MA, USA). After washing with 3 mL of water to isolate the polar fraction, the non-polar fraction was eluted using 2 mL of methanol and 1 mL of water and subsequently analyzed by HPLC. The brain tissue was collected and homogenized in a mixture of MeCN and H2O (50/50, 4 mL) before centrifugation (5 min, 4000 rpm, 20ºC, Hettich Universal 32). The obtained supernatant was separated from the precipitate and analyzed via HPLC. Analytical HPLC was performed using a Dionex UltiMate 3000 HPLC system equipped with Chromeleon software (version 6.8), utilizing a Gemini C18 5-µm (10 × 250 mm) column (Phenomenex, Torrance, CA, USA). All fractions were assessed for radioactivity using a Wizard Gamma counter 1470 or 2480 (Wallac/PerkinElmer, Waltham, MA, USA).

### Biodistribution

Wistar/RccHan (WIST) rats from Inotiv, the Netherlands, received intravenous injections via a tail vein catheter of either [^11^C]DPA-813 (30–50 MBq) or [^18^F]DPA-814 (20–30 MBq). The rats were euthanized at 5, 15, and 45 min post-injection (*n* = 4 per time point). Blood, heart, lungs, liver, spleen, kidney, urine, bone, brain, duodenum, small intestine, skin, and muscle were collected, weighed, and their radioactivity measured using a Wizard Gamma counter 1470 or 2480 (Wallac/PerkinElmer, Waltham, MA, USA). Entire organs were collected, except for larger organs like the liver and lungs, where only a portion was dissected. The radioactivity uptake in each organ was expressed as the percentage of the injected dose per gram of tissue (%ID/g).

### Human tissues and TSPO *rs6971* SNP determination

Brain tissues from MS patients (*n* = 7) were used in this study and were obtained from the MS centre of the Amsterdam University Medical Centers (AUMC). For some patient material, several brain tissues were available. The MS centre received permission from the Ethical Committee of the AUMC (Amsterdam, The Netherlands) to perform autopsies for tissue use and access to medical records for research purposes. All patients and controls, or their next of kin, had given informed consent for autopsy and use of brain tissue for research purposes.

TSPO *rs6971* SNP was determined with a Taqman assay (C_2512465_20, ThermoFisher Scientific, The Netherlands). DNA was isolated from human MS brain tissue cryosections using a QIAmp mini kit from Qiagen according to the manufacturer’s recommendation. The concentration of the isolated DNA was determined on a nanodrop (NanoVue Plus, Biochrom). DNA concentration ranged from 45 to 246 µg/mL. QPCR was performed on a QuantStudio (Applied Biosystems, The Netherlands). Synthetic DNA (300 bp) for the two TSPO SNP alleles, rs6971-A, and rs6971-G, was used as a control for the Taqman assay. DNA samples were diluted 1/100 in milliQ water, and 4.5 µL was used for the QPCR. Control DNA sequences were reconstituted in 10 ng/mL in milliQ water. The final QPCR solution contained 5 µL TaqPath proAmp master mix (ThermoFisher Scientific, The Netherlands), 0.5 µL Taqman SNP, and 4.5 µL DNA. Results of the Taqman assay are summarised in Supplementary Tables 4 and Supplementary Fig. 2.

Polymorphisms of *rs6971* within the TSPO gene, which causes an Ala to Thr substitution at position 147, were categorised as high-affinity binders (HABs; C/C DNA polymorphism; Ala/Ala at position 147), mixed affinity binders (MABs; C/T DNA polymorphism; Ala/Thr) or low-affinity binders (LABs; T/T DNA polymorphism; Thr/Thr).

### Autoradiography

Human MS tissue cryosections (20 μm) were mounted on glass slides, air-dried, and stored at − 80 ºC until needed. Prior to use, the sections were washed three times in PBS for 5 min each, then dried under an airflow. The sections were incubated with [^11^C]DPA-813, [^11^C]DPA-713, [^18^F]DPA-814, or [^18^F]DPA-714 (1-1.5 MBq per slide for ^11^C-labeled tracers and 0.2 MBq per slide for ^18^F-labeled tracers) in assay buffer (PBS + 1% cyclodextrin) at room temperature for 90 min. For blocking experiments, tissue sections were co-incubated with PK 11195 (10 µM) and the tracer. After incubation, sections were washed for 90 s three times each in ice-cold PBS and then dipped in deionized water twice. The sections were air-dried and exposed to a phosphor screen BAS-IP SR 2025 (General Electric, Eindhoven, The Netherlands). Phosphor screens were subsequently imaged using a Typhoon FLA 7000 imager (General Electric, Eindhoven, The Netherlands), and signal intensity was quantified using ImageLab software (BioRad, Lunteren, The Netherlands).

### PET imaging in the EAE model

PET imaging was conducted on EAE rats at the peak of clinical symptoms (days 11–14 post-immunization). CFA control animals were imaged at the same time frame of 11–14 days post-immunization. Both EAE and CFA groups were imaged with [^11^C]DPA-813 and [^11^C]DPA-713 (EAE: *n* = 5 rats; CFA: *n* = 3 rats) with a 4 to 24-hour interval between scans, or with [^18^F]DPA-814 and [^18^F]DPA-714 (EAE: *n* = 6 rats; CFA: *n* = 3 rats) with a 24 to 28-hour interval between scans to facilitate head-to-head comparison of the tracers. Dynamic PET imaging was acquired using small animal NanoPET/CT and NanoPET/MR scanners (Mediso Ltd., Budapest, Hungary) equipped with similar PET detectors. 2–4% isoflurane in oxygen (1 L/min) was used to anesthetize the animals. The rats were positioned on the scanner bed, and their respiratory rate was monitored throughout the scan, with adjustments to anesthesia as needed. PET tracers (19–24 MBq) were injected intravenously via a tail vein catheter, and PET scans were conducted for 60 min immediately following tracer administration. The scans were acquired in list mode and rebinned into the following frame sequences: 4 × 5s, 4 × 10s, 2 × 30s, 3 × 60s, 2 × 300s, 1 × 600s, 1 × 900s, and 1 × 1200s for [^11^C]DPA-813 and [^11^C]DPA-713; and 4 × 5s, 4 × 10s, 2 × 30s, 3 × 60s, 2 × 300s, 3 × 600s, and 1 × 900s for [^18^F]DPA-814 and [^18^F]DPA-714. A fully three-dimensional algorithm (Tera-TomoTM, Mediso Ltd.) with 4 iterations, 6 subsets, and an isotropic 0.4 mm voxel dimension was used fro imaging reconstruction. Static reconstruction was based on the 30 and 60-minute frames. Images were analyzed and quantified using VivoQuant software (Invicro, Boston, USA) with integrated brain atlas fitting CT and MRI scans. Regions of interest (ROIs) were manually delineated on the spinal cord and lung, and a brain atlas was applied to various brain regions. Data analysis was performed using GraphPad Prism 9 (San Diego, CA, USA).

### Statistical analysis

All data are expressed as mean ± SD unless otherwise noted. Comparisons between groups were conducted using the two-tailed Student’s t-test for normally distributed data and the Mann-Whitney or Wilcoxon tests for non-normally distributed data. Statistical analyses were performed using GraphPad Prism versions 9 and 10 (San Diego, CA, USA). A *p* value of less than 0.05 was deemed statistically significant.

## Results

### Synthesis and in vitro binding evaluation of novel TSPO ligands

Taking inspiration from second-generation TSPO PET tracers DPA-713 and DPA-714 (Table 1), we synthesised a library of novel pyrazolo[1,5-*a*]-pyrimidines with altered acetamide substitution patterns (see Supplementary Information for detailed procedures). In vitro binding affinities (K_i_) for human TSPO were initially assessed using a competition radioligand assay against [^3^H]PK 11195 in our well-characterised assay using membranes isolated from transfected HEK-293T cells overexpressing either WT or A147T TSPO [32]. This structure-activity relationship study will be reported in a separate publication. Two of these new analogues, DPA-813 and DPA-814, displayed single-digit nanomolar binding affinity for both TSPO isoforms. Chemical structures and binding affinities are displayed in Table 1 alongside experimentally derived lipophilicity values (logD_7.4_) and predicted CNS permeability scores for both compounds. Data for DPA-713 and DPA-714 are included for comparison. Importantly, for pre-clinical validation studies we showed that the new compounds also bound with high affinity to rat TSPO in competition with [^3^H]PK 11195 (Supplementary Fig. 12). Neither compound showed significant binding to 15 common off-target receptors found in the CNS (Supplementary Table 5).

**Table 1:**
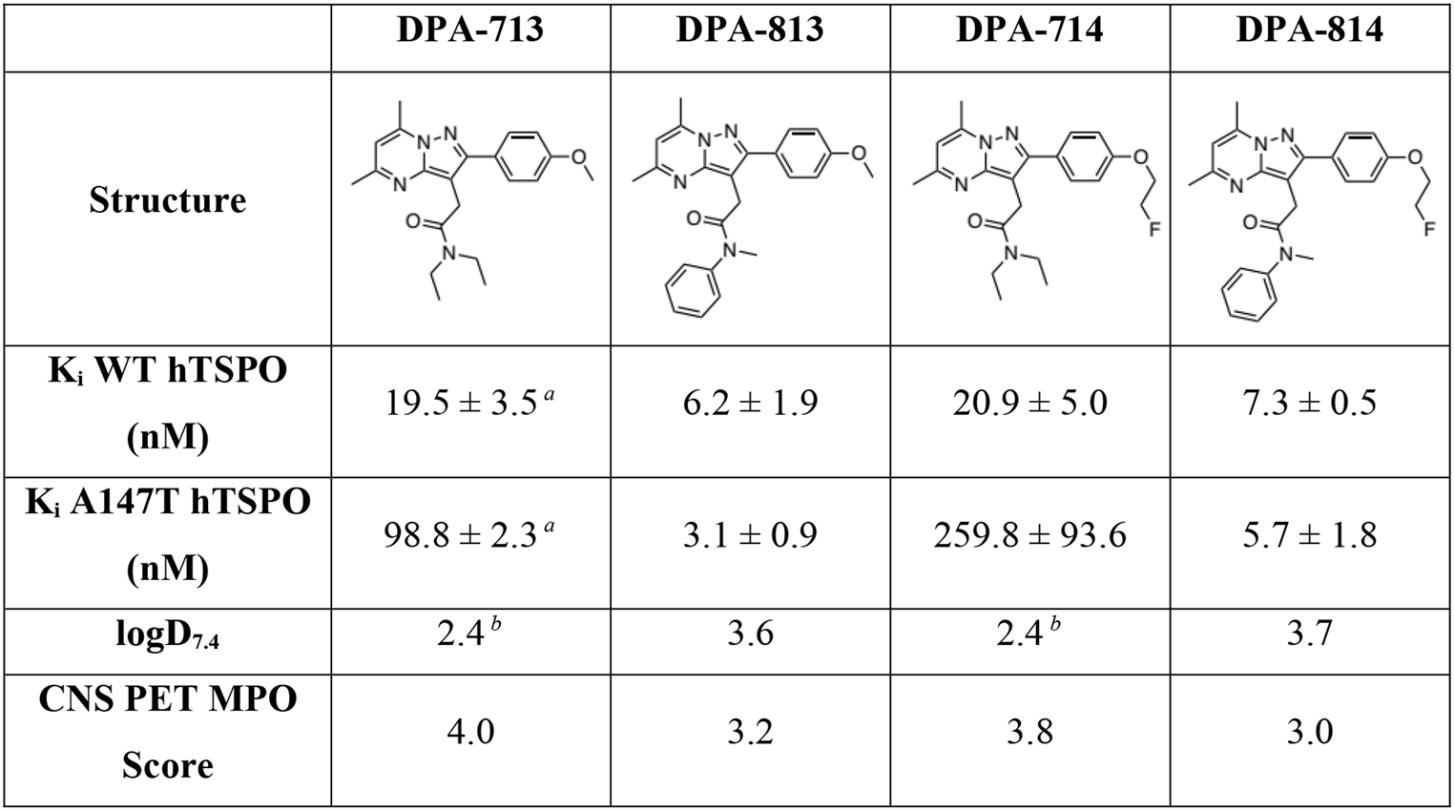
Chemical structures, in vitro binding affinities, lipophilicities, and predicted CNS permeability scores for second-generation TSPO ligands (DPA-713 & DPA-714) and novel, polymorphism insensitive TSPO ligands (DPA-813 & DPA-814). ^*A*^ K_i_ obtained from reference [32]; this was measured using identical assay conditions to all other compounds in table 1; details provided in materials and methods. ^*B*^ distribution coefficients obtained from reference [16]

### Radiochemistry

**Scheme 1:**
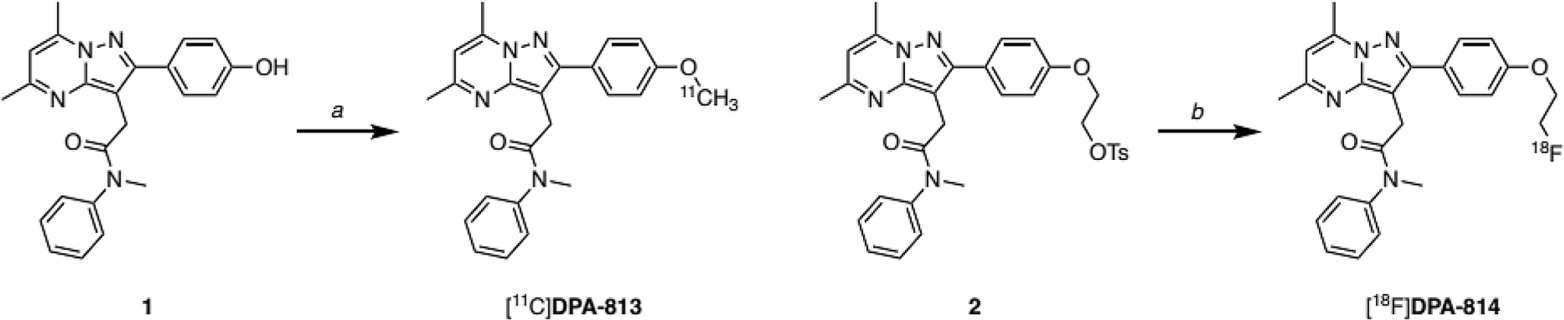
Radiolabelling of [^11^C]DPA-813 and [^18^F]DPA-814. Reagents and conditions: (**a**) [^11^C]CH_3_I, NaOH, DMF, 80 °C, 5 min; (**b**) [^18^F]fluoride, K[2.2.2], K_2_CO_3_, CH_3_CN, 105 °C, 10 min

Radiolabelling precursors 1 and 2 (Scheme 1) were synthesised using modified literature procedures (see Supplementary Information). Early installation of an *O*-isopropyl ether protecting group [31] and subsequent deprotection using methanesulfonic acid [36] enabled facile access to phenol 1– the precursor for synthesis of [^11^C]DPA-813. This divergent intermediate was alkylated to yield non-radioactive DPA-813 and DPA-814 as reference standards, as well as tosylate 2– the precursor for synthesis of [^18^F]DPA-814. The tracers, [^11^C]DPA-813 and [^18^F]DPA-814 (Scheme 1), were obtained with high purity (> 95%) and moderate molar activities (> 28 GBq/µmol and radiochemical yields (23–43%, corrected to end of bombardment)).

### Ex vivo biodistribution and metabolite analysis in rats

**Fig. 1:**
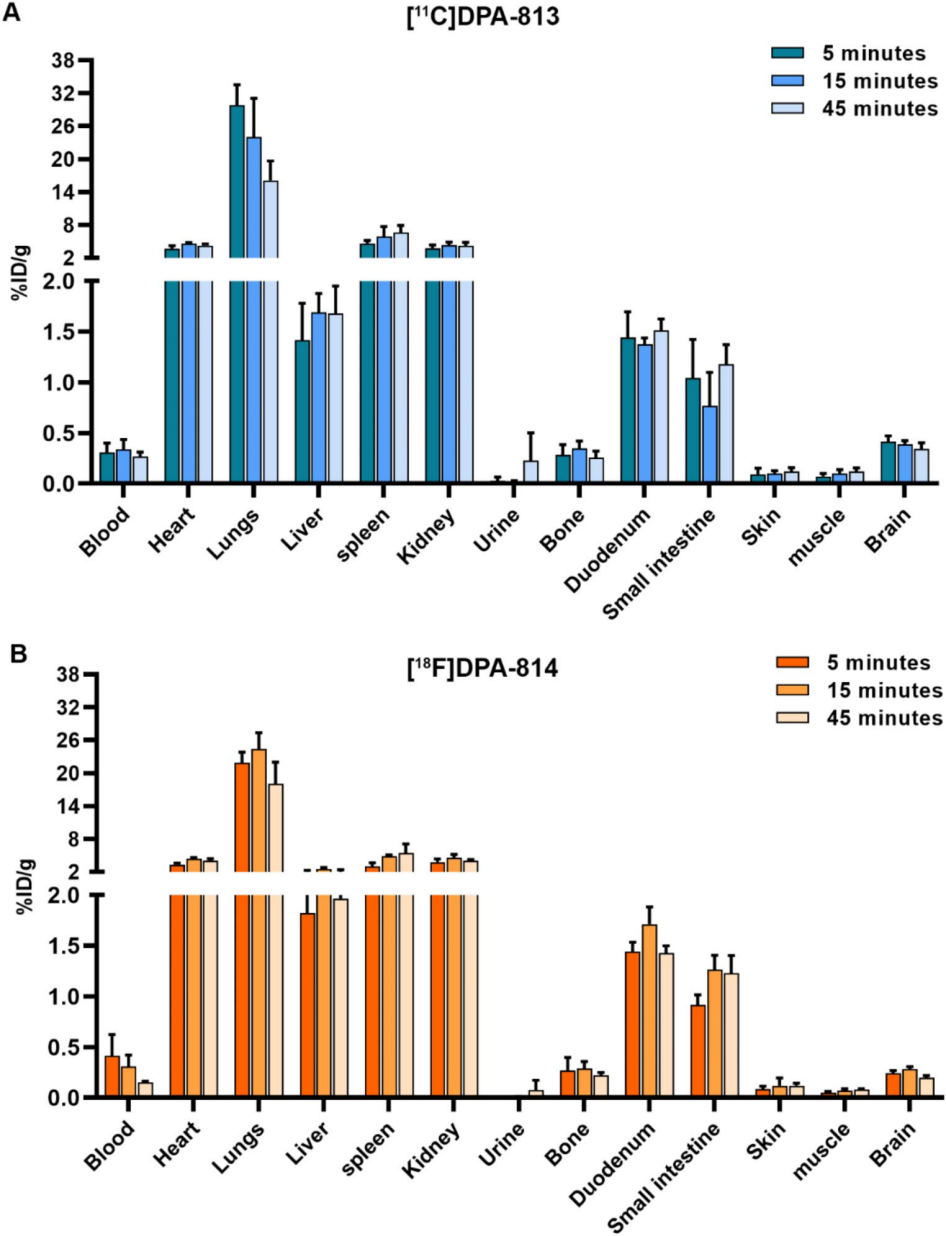
Ex vivo biodistribution analysis of [^11^C]DPA-813 and [^18^F]DPA-814 in wild-type rats. Biodistribution of [^11^C]DPA-813 **(A)** and [^18^F]DPA-814 **(B)** at 5, 15, and 45 min post-injection

The biodistribution of [^11^C]DPA-813 and [^18^F]DPA-814 was evaluated in WT rats at 3 different time points. Both tracers showed fast brain uptake with 0.41 ± 0.1%ID/g for [^11^C]DPA-813 and 0.25 ± 0.03%ID/g for [^18^F]DPA-814 at 5 min post-injection (p.i.), and slow washout over time with 0.34 ± 0.1%ID/g and 0.20 ± 0.02%ID/g for [^11^C]DPA-813 and [^18^F]DPA-814, respectively, at 45 min p.i. Blood kinetics were better for [^18^F]DPA-814 with faster clearance over time (0.41 ± 0.2%ID/g at 5 min to 0.15 ± 0.01%ID/g at 45 min) compared to [^11^C]DPA-813 (0.31 ± 0.1%ID/g at 5 min to 0.27 ± 0.01%ID/g at 45 min). Comparable moderate uptake was observed in the heart, spleen, kidneys, duodenum, and small intestine for both tracers, with minimal washout from these organs. Both tracers showed significant lung uptake with 29 ± 3.7%ID for [^11^C]DPA-813 and 21.91 ± 1.9%ID for [^18^F]DPA-814 at 5 min, and only minimal washout over time. Conversely, liver uptake was low for both tracers, peaking at 15 min p.i. with 1.69 ± 0.2%ID/g for [^11^C]DPA-813 and 2.56 ± 0.2%ID/g for [^18^F]DPA-814 (Fig. 1 and Supplementary Table 1).

**Fig. 2:**
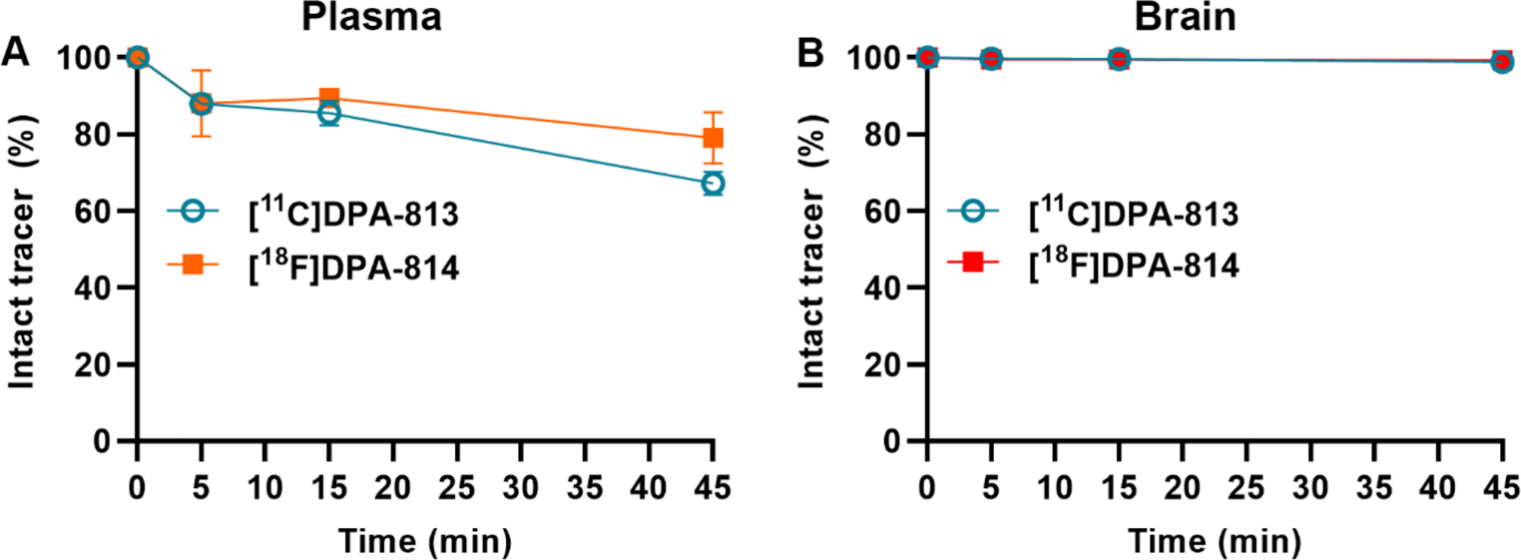
Ex vivo metabolite analysis of [^11^C]DPA-813 and [^18^F]DPA-814 in wild-type rats. Percentage of intact [^11^C]DPA-813 and [^18^F]DPA-814 in the plasma **(A)** and brain **(B)** at 5, 15, and 45 min post-injection

[^11^C]DPA-813 and [^18^F]DPA-814 demonstrated excellent plasma stability with 67.2 ± 2.9% and 79.1 ± 6.6% of intact tracer remaining after 45 min p.i., respectively. Both tracers appeared highly stable in the brain with more than 99% intact tracer after 45 min p.i. (Fig. 2, Supplementary Fig. 1, and Supplementary Table 2).

### Binding of [^11^C]DPA-813 and [^18^F]DPA-814 on MS tissues with WT and A147T TSPO

To validate the insensitivity of the [^11^C]DPA-813 and [^18^F]DPA-814 to the TSPO A147T SNP and confirm their superiority over the second-generation tracers [^11^C]DPA-713 and [^18^F]DPA-714, we evaluated their binding on human MS tissues by autoradiography. The presence of the A147T SNP was checked for all the MS tissues using a Taqman SNP test, and tissues were classified as HAB (WT/WT) (*n* = 4), MAB (WT/A147T) (*n* = 2), and LAB (A147T/A147T) (*n* = 1). For some MS patients, materials from different brain areas were available and used for the autoradiography studies. (Supplementary Tables 4 and Supplementary Fig. 2). MS tissues were also characterised for neuroinflammation and showed intensive microgliosis and microglial activation (Supplementary Fig. 3). The binding of [^11^C]DPA-813 and [^11^C]DPA-713, as well as the binding of [^18^F]DPA-814 and [^18^F]DPA-714, were assessed on adjacent tissue sections for comparison. Blocking studies were also performed with PK 11195 (10 µM) on the human MS tissue to validate the specific binding to TSPO (Supplementary Fig. 4A and B).

**Fig. 3:**
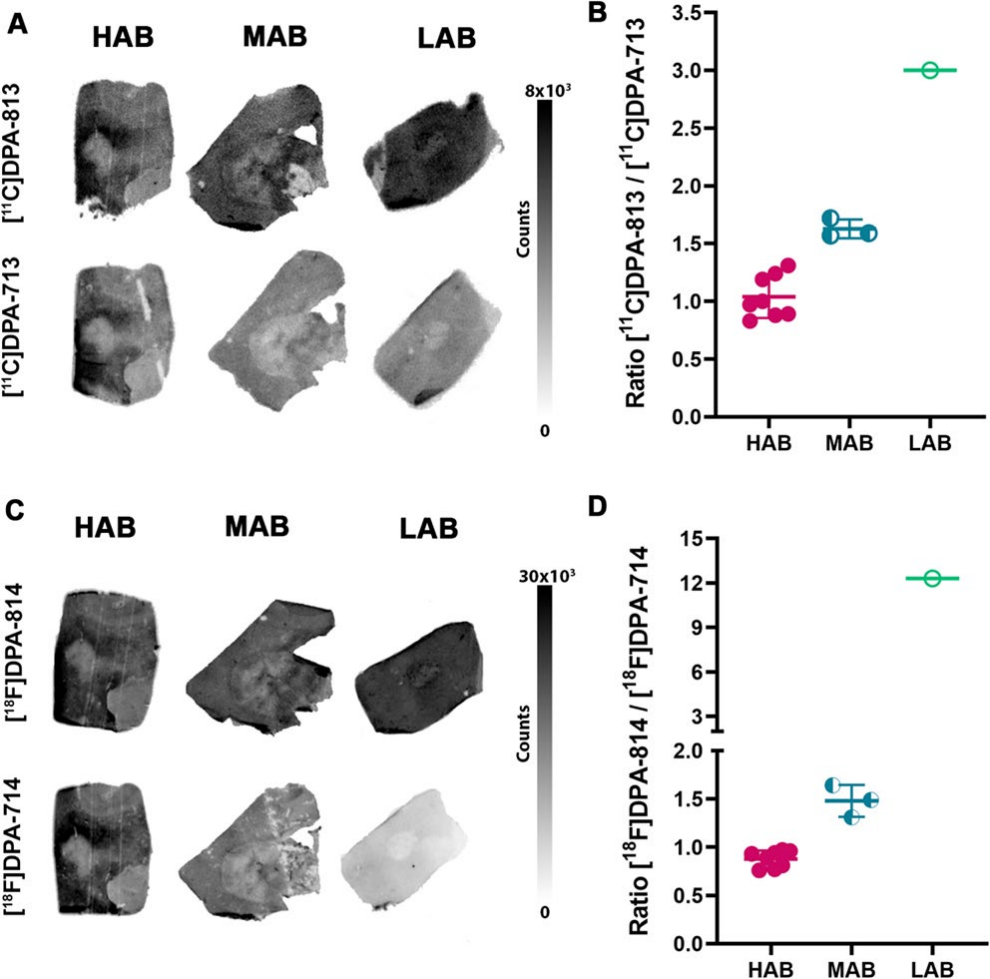
Autoradiography on high (HAB), mixed (MAB), and low (LAB) affinity binder human multiple sclerosis tissues. (**A**): Autoradiograph comparing the binding of [^11^C]DPA-813 and [^11^C]DPA-713 to HAB, MAB, and LAB. (**B**): Ratio of [^11^C]DPA-813 to [^11^C]DPA-713 binding. (**C**): Autoradiograph comparing the binding of [^18^F]DPA-814 and [^18^F]DPA-714 to HAB, MAB, and LAB. (**D**): Ratio of [^18^F]DPA-814 to [^18^F]DPA-714 binding. HAB, WML (Frontal gyrus / Periventricular lesion); MAB, WML (Medial frontal gyrus / Deep white matter); LAB, WML (Frontal gyrus / Deep white matter)

[^18^F]DPA-814 and [^11^C]DPA-813 showed high binding to the MS tissues irrespective of the TSPO genetic polymorphism (Fig. 3A, C). [^11^C]DPA-813 showed comparable binding to [^11^C]DPA-713 on HAB tissues, while on MAB tissues [^11^C]DPA-713 exhibited lower binding and significantly less binding on LAB tissue compared to [^11^C]DPA-813. The ratio of the binding quantification of [^11^C]DPA-813/ [^11^C]DPA713 was 1 ± 0.2 for HAB, 1.6 ± 0.1 for MAB, and 3 for LAB.

Similar trends were observed for the binding of [^18^F]DPA-714 compared to [^18^F]DPA-814, which displayed comparable binding levels on HAB, decreased binding on MAB, and nearly no binding on LAB tissue. The ratio of the binding quantifications of [^18^F]DPA-814/ [^18^F]DPA-714 was 0.9 ± 0.1 for HAB, 1.5 ± 0.2 for MAB, and 12.3 for LAB. Blocking studies with PK 11195 showed specific binding to TSPO for all the tracers with minimal non-specific binding (Supplementary Fig. 4A and B).

### PET imaging in the EAE rat model

**Fig. 4:**
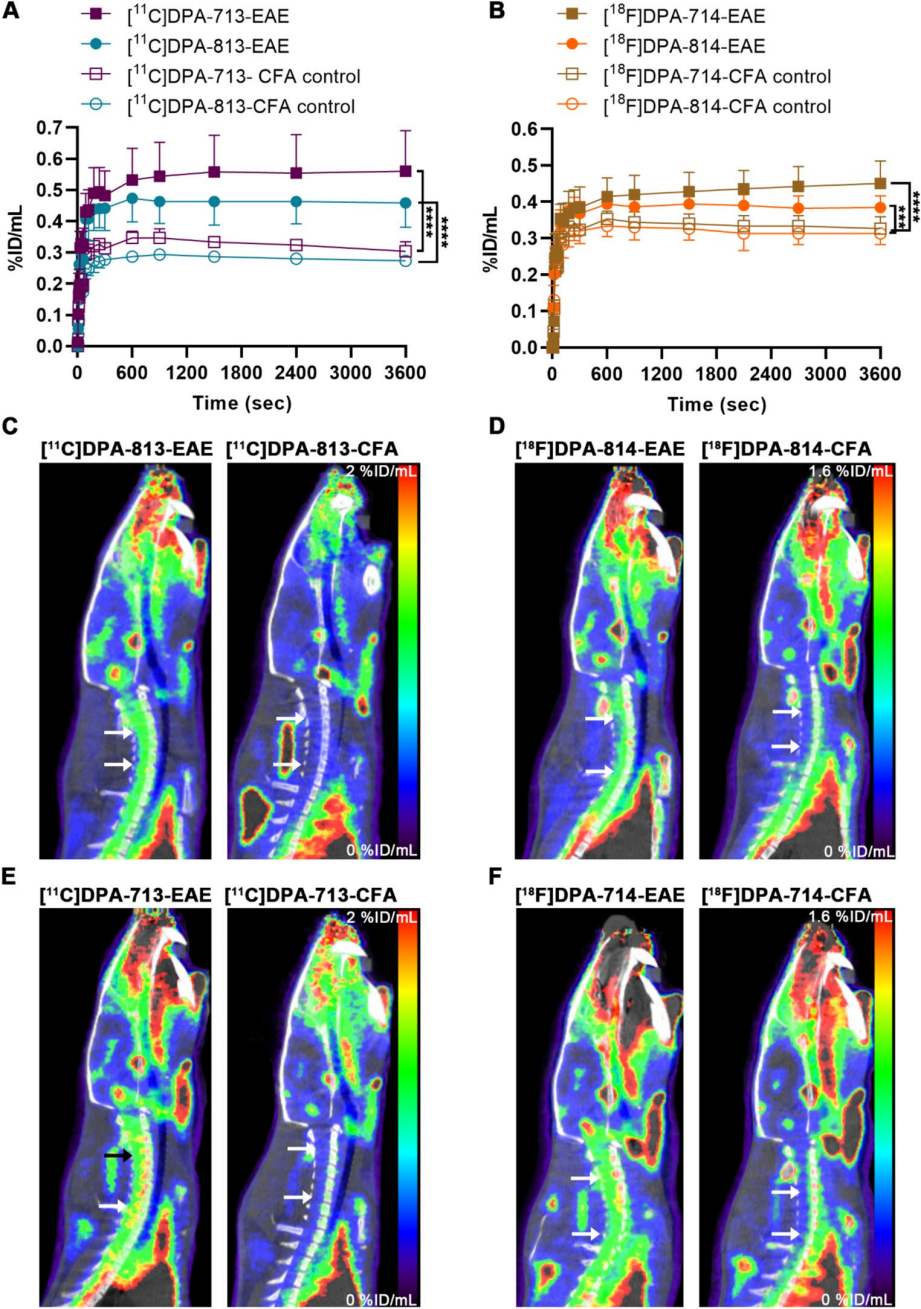
(**A**): Time activity curves (TAC) of [^11^C]DPA-813, [^11^C]DPA-713, and (**B**):[^18^F]DPA-814, and [^18^F]DPA-714. (**C**): Representative PET images showing the uptake in the spinal cord (black arrows) in EAE rats and CFA control for [^11^C]DPA-813, (**D**): [^18^F]DPA-814, (**E**): [^11^C]DPA-713 and (**F**): [^18^F]DPA-714. Data are expressed as percent injected dose per millilitre (%ID/mL) (***, *p* < 0.001; ****, *p* < 0.0001)

EAE in Lewis rats induces acute severe neuroinflammation in the CNS. To evaluate the potential of [^11^C]DPA-813 and [^18^F]DPA-814 for in vivo imaging of neuroinflammation and to compare their performance with [^11^C]DPA-713 and [^18^F]DPA-714, we conducted PET imaging with these tracers in the EAE model. Animals were imaged at the peak stage of neuroinflammation (maximum of clinical signs). [^11^C]DPA-813 and [^11^C]DPA-713 were imaged in the same animals with a 4–24 h difference between the scans. [^18^F]DPA-814 and [^18^F]DPA-714 were also imaged sequentially in the same animals with 24–28 h difference. Animals immunised with CFA (no neuroinflammation develops in these rats) were used as controls. In the acute EAE model, neuroinflammation manifests mainly in the spinal cord and, to a lesser extent, in the mid-brain and cerebellum (Supplementary Fig. 10). The spinal cord time activity curves for the different tracers in EAE and CFA animals are presented in Fig. 4A and B and Supplementary Fig. 9.

All four tracers showed rapid uptake in the spinal cord that plateaued around 15 min post-injection with minimal washout. [^11^C]DPA-813 showed significantly higher uptake in the spinal cord of the EAE rats compared to the CFA control (64% by comparing the area under the curve (AUC) of the groups, *p* < 0.0001). Similarly, [^11^C]DPA-713 demonstrated higher uptake in the spinal cord of the EAE rats compared to the CFA control (AUC 67% higher, *p* < 0.0001). Overall, [^11^C]DPA-813 showed lower uptake in the spinal cord compared to [^11^C]DPA-713 in both EAE and CFA; however, the difference in uptake between the EAE and CFA groups was comparable (Fig. 4A, C, and E).

For [^18^F]DPA-814, the uptake in the spinal cord of the EAE rats was higher compared to the CFA animals, with 21% higher AUC (*p* < *0.0001)*. [^18^F]DPA-714 also showed higher uptake in the spinal cord of the EAE rats compared to the CFA controls (AUC 27% higher, *p* < 0.001). Compared to [^18^F]DPA-714, [^18^F]DPA-814 exhibited lower uptake with smaller differences between the EAE and CFA groups (Fig. 4B, D, and F).

Quantifying static reconstruction of the different brain regions showed higher uptake in the mid-brain for all the tracers in the EAE animals compared to the CFA control. Similar trends with higher levels of uptake were observed for the [^11^C]DPA-713 and [^18^F]DPA-714 compared to [^11^C]DPA-813 and [^18^F]DPA-814, respectively (Supplementary Fig. 8).

Since notably high lung uptake was observed in the biodistribution of [^11^C]DPA-813 and [^18^F]DPA-814, we investigated this further by looking at their dynamic lung uptake. [^11^C]DPA-813 and [^18^F]DPA-814 demonstrated fast and high lung uptake that plateaued after 5 min p.i. with minimal washout over time. In comparison, [^11^C]DPA-713 and [^18^F]DPA-714 had a high initial peak within a couple of minutes p.i. and very fast washout from the lungs (Supplementary Fig. 7).

## Discussion

As part of our tracer discovery campaign to develop polymorphism-insensitive TSPO PET tracers, we synthesised a series of novel analogues of known, pyrazolo[1,5-a]pyrimdine based, second-generation TSPO ligands DPA-713 [15] and DPA-714 [16] with modified acetamide substitution patterns [37]. A detailed summary of our extensive structure-activity relationship study will be reported separately in due course. It has previously been shown that changing *N*-substituents at these positions influences ligand binding to rat TSPO, but no systematic investigation of binding to human WT and A147T TSPO has been reported [38, 39]. Two of our novel compounds incorporated *N*-methyl-*N*-phenylacetamides in place of *N*,*N*-diethylacetamides common to the lead compounds. This structural alteration resulted in substantially increased binding affinity to both isoforms of human TSPO, but was especially pronounced for A147T (Table 1).

DPA-813 bound with high affinities of 6.2 nM and 3.1 nM to WT and A147T TSPO, respectively. This represented a modest ~ 3-fold increase in affinity to the WT, but a substantial ~ 32-fold increase to A147T versus parent compound DPA-713. Fluorinated analogue DPA-814 displayed similar affinities of 7.3 nM (WT) and 5.7 nM (A147T), representing 3-fold and 46-fold increases in affinity to WT and A147T TSPO, respectively, compared with DPA-714. Our new compounds also significantly outperformed (*R*)-PK 11195 (WT K_i_ = 29.2, A147T K_i_ = 36.0 nM in our assay) [32]. These results represent the first evidence that modifying the acetamide moiety of pyrazolo[1,5-*a*]pyrimidine-based ligands can enhance affinity for A147T TSPO. This contrasts with our previous SAR study where similar modifications generally resulted in reduced binding affinity for this isoform [40]. 

Despite their potential as polymorphism-insensitive TSPO PET tracers, it was important to evaluate the binding affinity of these new compounds to rat TSPO prior to radiolabelling and in vivo PET studies. Our prior studies have shown that DPA-713 and DPA-714 bind with higher affinity to rat TSPO than WT human TSPO [30, 32]. DPA-813 and DPA-814 were also observed to follow this trend, each binding to rat TSPO with subnanomolar affinity (Supplementary Fig. 12). It should be noted that rats and other laboratory animals do not possess a spontaneous TSPO mutation analogous to human polymorphism *rs6971*.

Our novel compounds exhibited increased lipophilicities (logD_7.4_) versus the parent compounds, but calculated CNS PET MPO scores predicted a high likelihood of successful brain uptake (Table 1) [34]. Therefore, we elected to synthesise carbon-11 and fluorine-18 radiolabelled analogues of DPA-813 and DPA-814, respectively, to further investigate their potential as polymorphism insensitive TSPO PET tracers (Scheme 1). As a next step, we performed autoradiography with the radiolabeled novel compounds on MS brain tissues characterized for pathology and A147T TSPO SNP. [^11^C]DPA-813 and [^18^F]DPA-814 presented high specific binding on all MS tissue irrespective of the TSPO polymorphism status. Specifically, on the tissue with LAB TSPO, the binding was 3- and 12-fold higher, respectively, compared to the parent compounds that showed mediocre tissue binding, validating the superiority of [^11^C]DPA-813 and [^18^F]DPA-814 and their insensitivity to the A147T TSPO SNP (Fig. 3). Additionally, higher binding of [^11^C]DPA-813 and [^18^F]DPA-814 on MS tissue correlated with the area containing lesions and glial activation, showing their specificity for targeting neuroinflammation (Supplementary Fig. 3).

A comparable metabolic stability profile was observed for [^11^C]DPA-813 and [^18^F]DPA-814, along with high brain stability and no brain-penetrant metabolites. Both compounds display good plasma stability (67% and 79% intact tracer at 45 min for ^11^C and ^18^F labeled compounds, respectively), which is higher than the parent compounds [41, 42]. [^11^C]DPA-813 and [^18^F]DPA-814 exhibited similar organ biodistribution with good brain uptake that was retained over time, likely due to the high affinity to rat TSPO and high stability of the tracers. Liver uptake was remarkably low despite the higher lipophilicity of the novel compounds that would suggest liver accumulation (Fig. 1). Lungs had particularly high uptake for both tracers with minimal washout. This was also observed with PET imaging in the EAE and control animals, where lung uptake hit a maximum shortly after injection and plateaued. This behavior was contrary to the [^11^C]DPA-713 and [^18^F]DPA-714, which showed initial peak uptake in the lungs that washed out with time (Supplementary Fig. 7). This higher lung retention may be due to increased lipophilicity of the [^11^C]DPA-813 and [^18^F]DPA-814 compared to the parent compounds.

The acute EAE model in Lewis rats is characterized by microglia activation and macrophage infiltration mainly in the spinal cord and, to a lesser extent, in the midbrain and cerebellum during the clinical phase [43, 44]. All the tracers displayed similar brain distribution in the EAE-driven inflammatory regions and TSPO-rich areas (the choroid plexus and the ventricular system) [45]. Both [^11^C]DPA-813 and [^18^F]DPA-814 showed high uptake in the spinal cord (Fig. 4) and the midbrain (Supplementary Fig. 8) compared to the control. While a blocking or displacement study could have provided additional insights into specific binding during PET imaging experiments, the uptake correlates well with immunohistochemical staining for activated microglia in the spinal cord of EAE and control rats post (Supplementary Fig. 10), validating their potential to image neuroinflammation in vivo. However, the maximum uptake in the spinal cord was slightly lower compared to the parent compounds in the EAE and control animals (Fig. 4). This can be the consequence of the higher affinity of [^11^C]DPA-813 (24-fold) and [^18^F]DPA-814 (35-fold) to the rat TSPO compared to the parent compounds, which can lead to less plasma availability of the tracer due to faster and higher depletion by TSPO expressed on peripheral immune cells [46]. Additionally, the higher lung uptake exhibited by the tracers can amplify this effect, leading to even lower plasma availability.

[^11^C]DPA-813 and [^11^C]DPA-713 performed better in vivo than the fluorinated compounds, with higher maximum uptake in the EAE animals and higher differences compared to the control animals (3 and 2.5 folds, respectively). A possible explanation can be related to a difference in brain permeability related to the fluorination and the faster blood clearance in the case of [^18^F]DPA-814 compared to [^11^C]DPA-813 (Fig. 1).

## Conclusions

We have successfully discovered and evaluated two new PET tracers, [^11^C]DPA-813 and [^18^F]DPA-814, that bind equally well to human WT and A147T TSPO, as demonstrated in cells and by autoradiography on post-mortem human brain tissue. PET studies in rats revealed good brain uptake and acceptable rate of metabolism, while in the EAE model increased uptake was observed in inflamed brain regions, comparable with that of clinically-used TSPO PET tracers [^11^C]DPA-713 and [^18^F]DPA-714. [^11^C]DPA-813 and [^18^F]DPA-814 could therefore be used for TSPO PET in humans of all genotypes, addressing a significant limitation of second-generation ligands, although the high retention in the lungs needs to be assessed carefully. Evaluation in head-to-head studies in patients versus healthy controls, with benchmarking against second-generation tracers, will further validate the sensitivity and potential clinical utility of these tracers.

## Electronic supplementary material

Below is the link to the electronic supplementary material.

Supplementary Material 1

## Data Availability

All data generated during and/or analysed during the current study are available either within the associated Supplementary Information document or from the corresponding authors on reasonable request.

## Abbreviations

Ala: Alanine
AUC: Area under the curve
AUMC: Amsterdam University Medical Centers
CNS: Central nervous system
CFA: Complete Freund’s adjuvant
EAE: Experimental autoimmune encephalomyelitis
HAB: High-affinity binder
LAB: Low-affinity binder
MS: Multiple Sclerosis
MAB: Moderate-affinity binder
MPO: Multiparameter Optimization
PET: Positron emission tomography
PBS: Phosphate buffer solution
QPCR: Quantitative polymerase chain reaction
ROI: Region of interest
SNP: Single nucleotide polymorphism
TAC: Time activity curve
TSPO: Translocator protein 18 kDa
Thr: Threonine
WT: Wild-type
%ID/g: Percent injected dose per gram
%ID/mL: Percent injected dose per millilitre
