# Supplementary material for: Development and evaluation of [11C]DPA-813 and [18F]DPA-814: novel TSPO PET tracers insensitive to human single nucleotide polymorphism rs6971
Source: Eur J Nucl Med Mol Imaging. 2025 Feb 5;52(7):2658–70. doi: 10.1007/s00259-025-07109-1 (PMC12119672; doi:10.1007/s00259-025-07109-1)
Supplement: Supplementary file 1 — Supplementary Material 1 [file 259_2025_7109_MOESM1_ESM.docx]

**Supplementary Information**

**Development and evaluation of [^11^C]DPA-813 and [^18^F]DPA-814: novel TSPO PET tracers insensitive to human single nucleotide polymorphism rs6971**

[Biodistribution 2](#_Toc160811917)

[Metabolite analysis 3](#_Toc160811918)

[Determination of the rs6971 TSPO SNP 4](#_Toc160811919)

[Autoradiography on Human MS tissue 7](#_Toc160811920)

[PET imaging in the EAE rat model 9](#_Toc160811921)

[Ex vivo validation of microglia activation in the spinal cord 12](#_Toc160811922)

[In vitro off-target screening 13](#_Toc160811923)

[General Synthesis Experimental 14](#_Toc160811924)

[Synthesis of DPA-813 and DPA-814 standards and radiolabelling precursors 15](#_Toc160811925)

[NMR Spectra of novel compounds 20](#_Toc160811926)

[HPLC Purity Analysis of Precursors and Standards 27](#_Toc160811927)

[Radioligand Binding Curves 31](#_Toc160811928)

[References 33](#_Toc160811929)

# Biodistribution

**Supplementary Table 1.** Biodistribution of [^11^C]DPA-813 and [^18^F]DPA-814 in wild type rats. Data is expressed as precent injected dose per gram (%ID/g).

|  | **[^11^C]DPA-813** | | | **[^18^F]DPA-814** | | |  |
| --- | --- | --- | --- | --- | --- | --- | --- |
|  | **5 min** | **15 min** | **45 min** | **5 min** | **15 min** | **45 min** | |
| **Blood** | 0.31±0.1 | 0.34±0.1 | 0.27±0.04 | 0.41±0.2 | 0.31±0.1 | 0.15±0.0 | |
| **Heart** | 3.70±0.5 | 4.57±0.2 | 4.28±0.3 | 3.31±0.3 | 4.39±0.2 | 4.05±0.3 | |
| **Lungs** | 29.84±3. | 24.03±7. | 16.13±3.5 | 21.91±1. | 24.43±2. | 18.05±3. | |
| **Liver** | 1.42±0.4 | 1.69±0.2 | 1.68±0.3 | 1.82±0.5 | 2.56±0.2 | 1.97±0.4 | |
| **spleen** | 4.62±0.6 | 5.88±1.8 | 6.66±1.2 | 3.00±0.6 | 4.85±0.2 | 5.45±1.6 | |
| **Kidney** | 3.80±0.5 | 4.31±0.5 | 4.20±0.6 | 3.81±0.5 | 4.59±0.6 | 4.10±0.2 | |
| **Urine** | 0.03±0.0 | 0.02±0.0 | 0.23±0.3 | 0.01±0.0 | 0.01±0.0 | 0.08±0.1 | |
| **Tail** | 1.30±0.6 | 1.18±0.1 | 1.33±0.7 | 6.89±9.8 | 0.70±0.2 | 1.30±0.5 | |
| **Bone** | 0.28±0.1 | 0.35±0.1 | 0.26±0.1 | 0.27±0.1 | 0.29±0.1 | 0.22±0.0 | |
| **Brain** | 0.41±0.1 | 0.39±0.0 | 0.34±0.1 | 0.25±0.0 | 0.28±0.0 | 0.20±0.0 | |
| **Duodenum** | 1.44±0.2 | 1.37±0.1 | 1.51±0.1 | 1.44±0.1 | 1.71±0.2 | 1.43±0.1 | |
| **Small intestine** | 1.04±0.4 | 0.77±0.3 | 1.18±0.2 | 0.92±0.1 | 1.26±0.1 | 1.23±0.2 | |
| **Skin** | 0.09±0.1 | 0.10±0.0 | 0.12±0.04 | 0.09±0.0 | 0.12±0.1 | 0.12±0.0 | |
| **muscle** | 0.07±0.0 | 0.10±0.0 | 0.12±0.03 | 0.05±0.0 | 0.07±0.0 | 0.08±0.0 | |

# Metabolite analysis


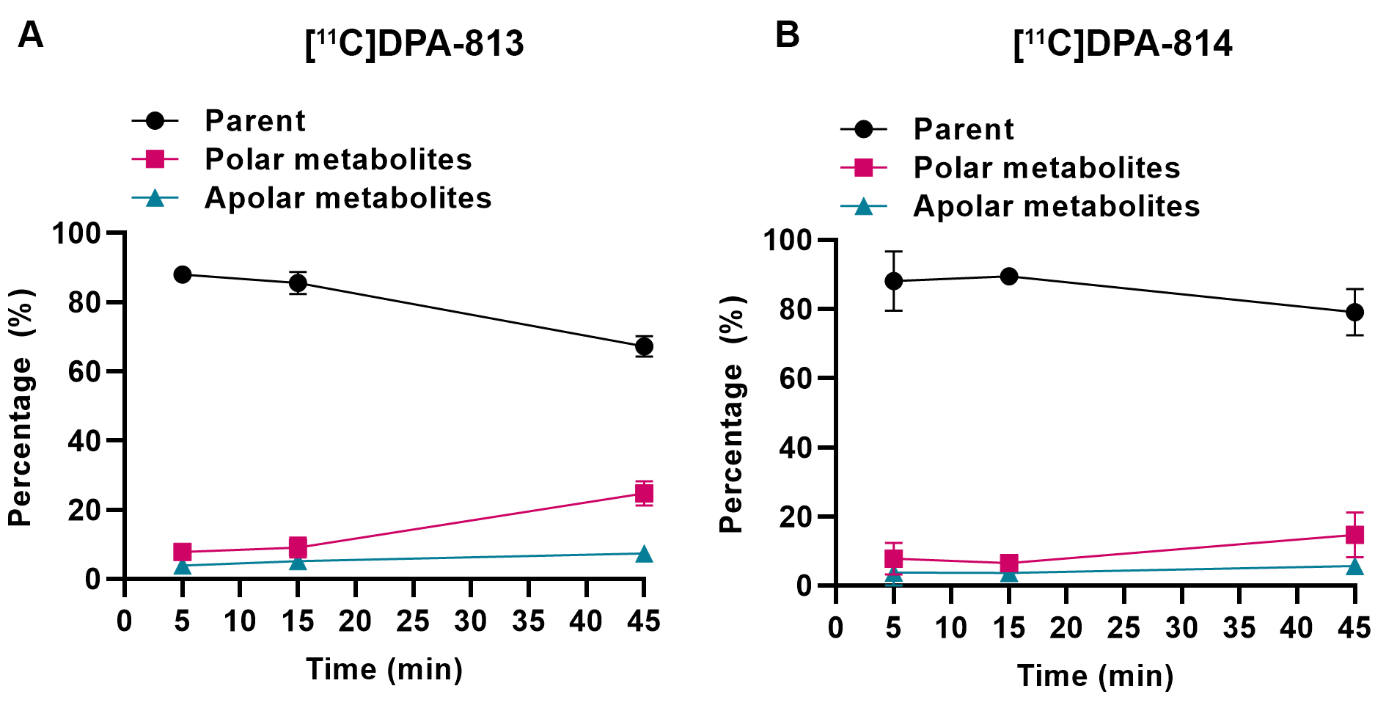


**Supplementary Figure 1.** *Ex vivo* metabolite analysis of [^11^C]DPA-813 **(A)** and [^18^F]DPA-814 **(B)** in wild-type rats. Percentage of parent, polar and apolar metabolites of [^11^C]DPA-813 and [^18^F]DPA-814 in the plasma at 5, 15, and 45 min post-injection.

**Supplementary Table 2.** Plasma and brain metabolite analysis results of [^11^C]DPA-813 and [^18^F]DPA-814 in wild-type rats

|  | **[^11^C]DPA-813 - Plasma** | | | **[^11^C]DPA-813 - Brain** |
| --- | --- | --- | --- | --- |
|  | **Parent (Intact tracer) (%)** | **Polar metabolites (%)** | **Apolar metabolites (%)** | **Parent (intact tracer) (%)** |
| **5 min** | 87.9 ± 2.4 | 7.8 ± 1.5 | 3.8 ± 1.5 | 99.7 ± 0.1 |
| **15 min** | 85.5 ± 3.2 | 9.1 ± 2.9 | 5.1 ± 1.7 | 99.6 ± 0.3 |
| **45 min** | 67.2 ± 2.9 | 24.7 ± 3.5 | 7.4 ± 1.9 | 98.9 ± 0.9 |
|  |  |  |  |  |
|  | **[^18^F]DPA-814 - Plasma** | | | **[^18^F]DPA-814 - Brain** |
|  | **Parent (Intact tracer) (%)** | **Polar metabolites (%)** | **Apolar metabolites (%)** | **Parent (intact tracer) (%)** |
| **5 min** | 88.1 ± 8.5 | 7.7 ± 4.6 | 3.7 ± 3.7 | 99.5 ± 0.2 |
| **15 min** | 89.4 ± 0.9 | 6.4 ± 1.2 | 3.6 ± 0.6 | 99.5 ± 0.3 |
| **45 min** | 79.1 ± 6.6 | 14.6 ± 6.4 | 5.6 ± 0.8 | 99.3 ± 0.3 |

# Determination of the *rs6971* TSPO SNP

**Supplementary Table 3.** MS patient tissues

| **Patient ID** | **Age** | **Gender** | **Years of MS** | **Type of MS** | **Delay post-mortem** | **Lesion type** | **Brain tissue region** |
| --- | --- | --- | --- | --- | --- | --- | --- |
| **Patient 1** | 59 | F | 22 | Progressive MS | 8h 50min | WML | Hippocampus |
|  |  |  |  |  |  | NAWM | Frontal lobe |
| **Patient 2** | 48 | F | 24 | Secondary progressive | 9h 20 min | WML | Frontal gyrus / Periventricular lesion |
| **Patient 3** | 75 | M | 14 | Secondary progressive | 9h 25 min | WML | Parietal lobe / Deep white matter |
|  |  |  |  |  |  | NAWM | Frontal gyrus / Deep white matter |
| **Patient 4** | 63 | F | 24 | Progressive MS | 10h 50 min | WML | Gyrus cingulate inferior |
| **Patient 5** | 51 | F | 17 | Secondary progressive MS | 9h 10 min | WML | Frontal gyrus / Deep white matter |
| **Patient 6** | 56 | M | 20 | Primary progressive MS | 6h 15min | WML | Inferior parietal lobe |
|  |  |  |  |  |  | WML | Gyrus cingulate |
|  |  |  |  |  |  | WML | Inferior parietal lobe / periventricular |
|  |  |  |  |  |  | WML | Inferior frontal gyrus / deep white matter |
| **Patient 7** | 67 | F | 29 | - | 11h 25 min | WML | Medial frontal gyrus / deep white matter |

*WML, white matter lesion; NAWM, normal appearing white matter; M, male; F, female*

**Sequence of synthetic control DNA for the WT (rs6971-A) and mutated (rs6971-G) TSPO gene**

**rs6971-A:** TGGGTCAGGTGGCATGACTGTTCCCATTTTACAGATGAGGAAACTGAGGCTGCGATGGGGGAGGGGCTTGGCCAGGTCACTCAAGGGTGGAGTGGGGGTGAGTGAGGCTCCTGACTCCCAAATCCAGTGGGAGTTGGGCAGTGGGACAGGCACTTGGGTGAACGCGGTGCCTCAGGCCTCCCCATCCTCCGTCCCCCAATCTCTGCAGGCCTTGGTGGATCTCCTGCTGGTCAGTGGGGCGGCGGCAGCCACTACCGTGGCCTGGTACCAGGTGAGCCCGCTGGCCGCCCGCCTGCTCTACCCCTACCTGGCCTGGCTGGCCTTC**A**CGACCACACTCAACTACTGCGTATGGCGGGACAACCATGGCTGGCGTGGGGGACGGCGGCTGCCAGAGTGAGTGCCCGGCCCACCAGGGACTGCAGCTGCACCAGCAGGTGCCATCACGCTTGTGATGTGGTGGCCGTCACGCTTTCATGACCACTGGGCCTGCTAGTCTGTCAGGGCCTTGGCCCAGGGGTCAGCAGAGCTTCAGAGGTGGCCCCACCTGAGCCCCCACCCGGGAGCAGTGTCCTGTGCTTTCTGCATGCTTAGAGCATGTTCTTGGAACATGGAATTTTATAAGCTGAATAAAGTTTTTGACTTCCTTTA

**rs6971-G:**

TGGGTCAGGTGGCATGACTGTTCCCATTTTACAGATGAGGAAACTGAGGCTGCGATGGGGGAGGGGCTTGGCCAGGTCACTCAAGGGTGGAGTGGGGGTGAGTGAGGCTCCTGACTCCCAAATCCAGTGGGAGTTGGGCAGTGGGACAGGCACTTGGGTGAACGCGGTGCCTCAGGCCTCCCCATCCTCCGTCCCCCAATCTCTGCAGGCCTTGGTGGATCTCCTGCTGGTCAGTGGGGCGGCGGCAGCCACTACCGTGGCCTGGTACCAGGTGAGCCCGCTGGCCGCCCGCCTGCTCTACCCCTACCTGGCCTGGCTGGCCTTC**G**CGACCACACTCAACTACTGCGTATGGCGGGACAACCATGGCTGGCGTGGGGGACGGCGGCTGCCAGAGTGAGTGCCCGGCCCACCAGGGACTGCAGCTGCACCAGCAGGTGCCATCACGCTTGTGATGTGGTGGCCGTCACGCTTTCATGACCACTGGGCCTGCTAGTCTGTCAGGGCCTTGGCCCAGGGGTCAGCAGAGCTTCAGAGGTGGCCCCACCTGAGCCCCCACCCGGGAGCAGTGTCCTGTGCTTTCTGCATGCTTAGAGCATGTTCTTGGAACATGGAATTTTATAAGCTGAATAAAGTTTTTGACTTCCTTTA

**Supplementary Table 4.** TSPO polymorphism results

| **Sample ID** | **Alleles** | **Affinity classification** |
| --- | --- | --- |
| Patient 1 | G/G | HAB |
| Patient 2 | G/G | HAB |
| Patient 3 | G/A | MAB |
| Patient 4 | G/G | HAB |
| Patient 5 | A/A | LAB |
| Patient 6 | G/G | HAB |
| Patient 7 | G/A | MAB |

**
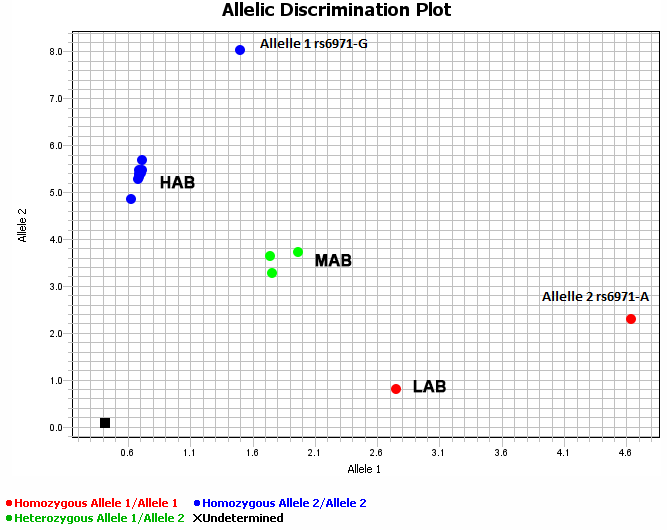
**

**Supplementary Figure 2.** Allelic discrimination plot showing the QPCR results of the Taqman rs6971 SNP test

# Autoradiography on Human MS tissue


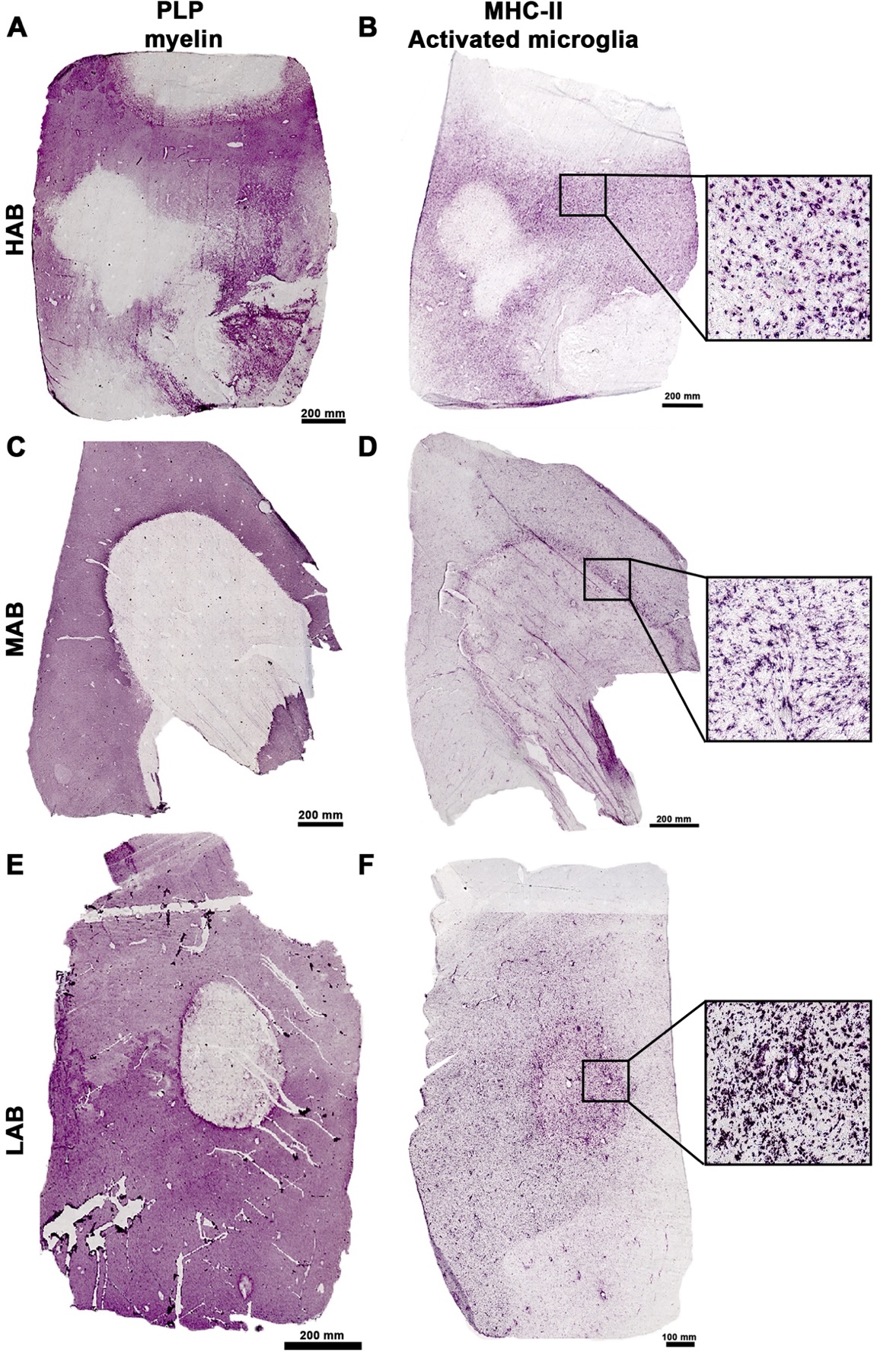
**Supplementary Figure 3.** Characterization of the MS human tissue for demyelination and activated microglia. Representative immunostaining images showing the PLP (myelin) and MHC-II (activated microglia) staining in representative MS tissues for the HAB, MAB, and LAB. PLP staining in HAB **(A)**, MAB **(C)** and LAB **(E)**. MHC-II staining in HAB **(B)**, MAB **(D)** and LAB **(F)**. Zoom images are shown for the MHC-II staining. HAB, WML (Frontal gyrus / Periventricular lesion); MAB, WML (Medial frontal gyrus / deep white matter); LAB, WML (Frontal gyrus / Deep white matter).


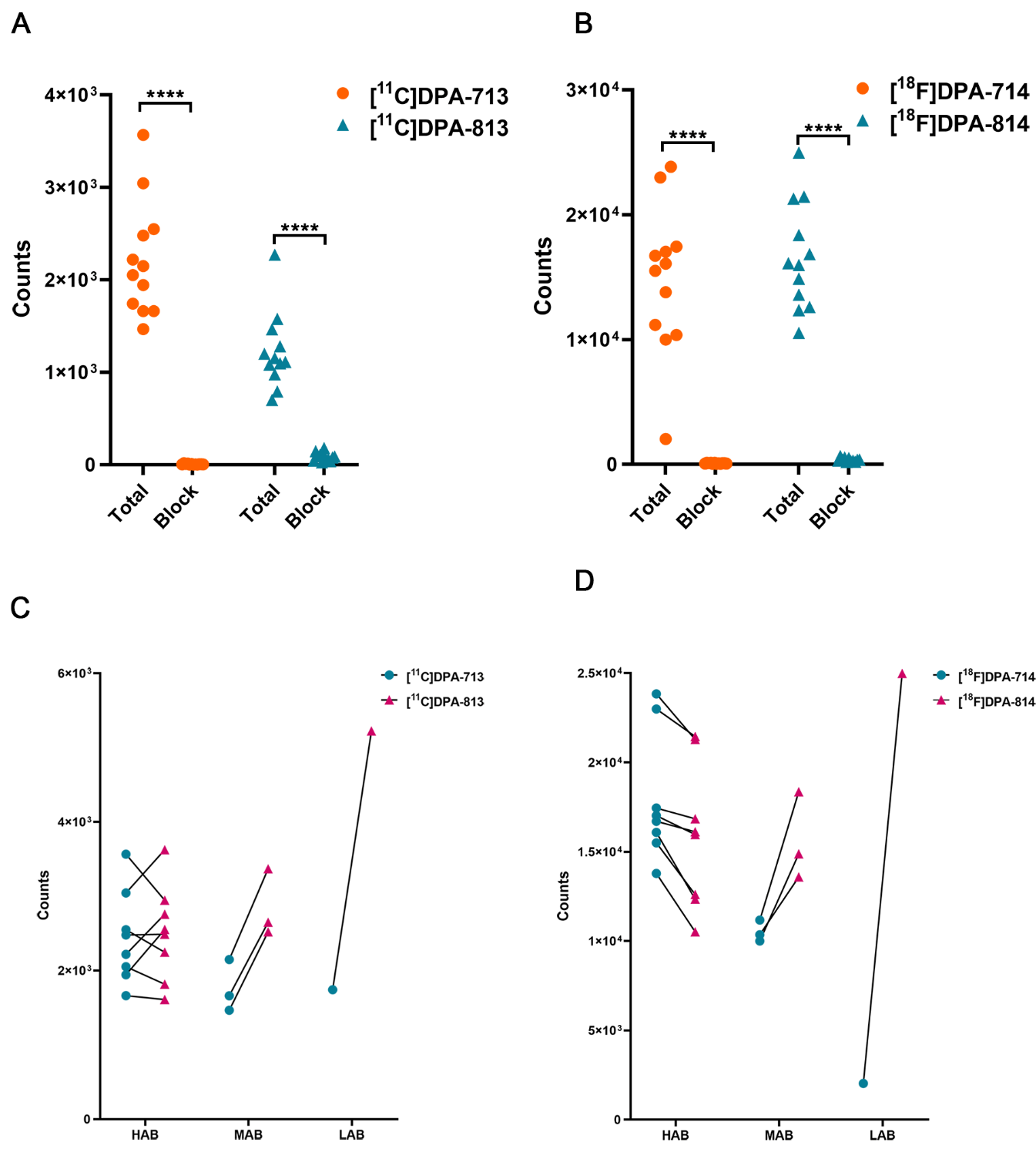


**Supplementary Figure 4.** Autoradiography on high (HAB), mixed (MAB), and low (LAB) affinity binder human multiple sclerosis tissues. Autoradiograph quantification of the binding of [^11^C]DPA-813 and [^11^C]DPA-713 with and without blocking with PK 11195 (10 µM) **(A)**, and [^18^F]DPA-814 and [^18^F]DPA-714 with and without blocking with PK 11195 (10 µM) **(B)**. Plots comparing the quantification of [^11^C]DPA-813 and [^11^C]DPA-713 **(C)** and [^18^F]DPA-814 and [^18^F]DPA-714 **(D)** binding to HAB, MAB and LAB human tissues.

#

# PET imaging in the EAE rat model


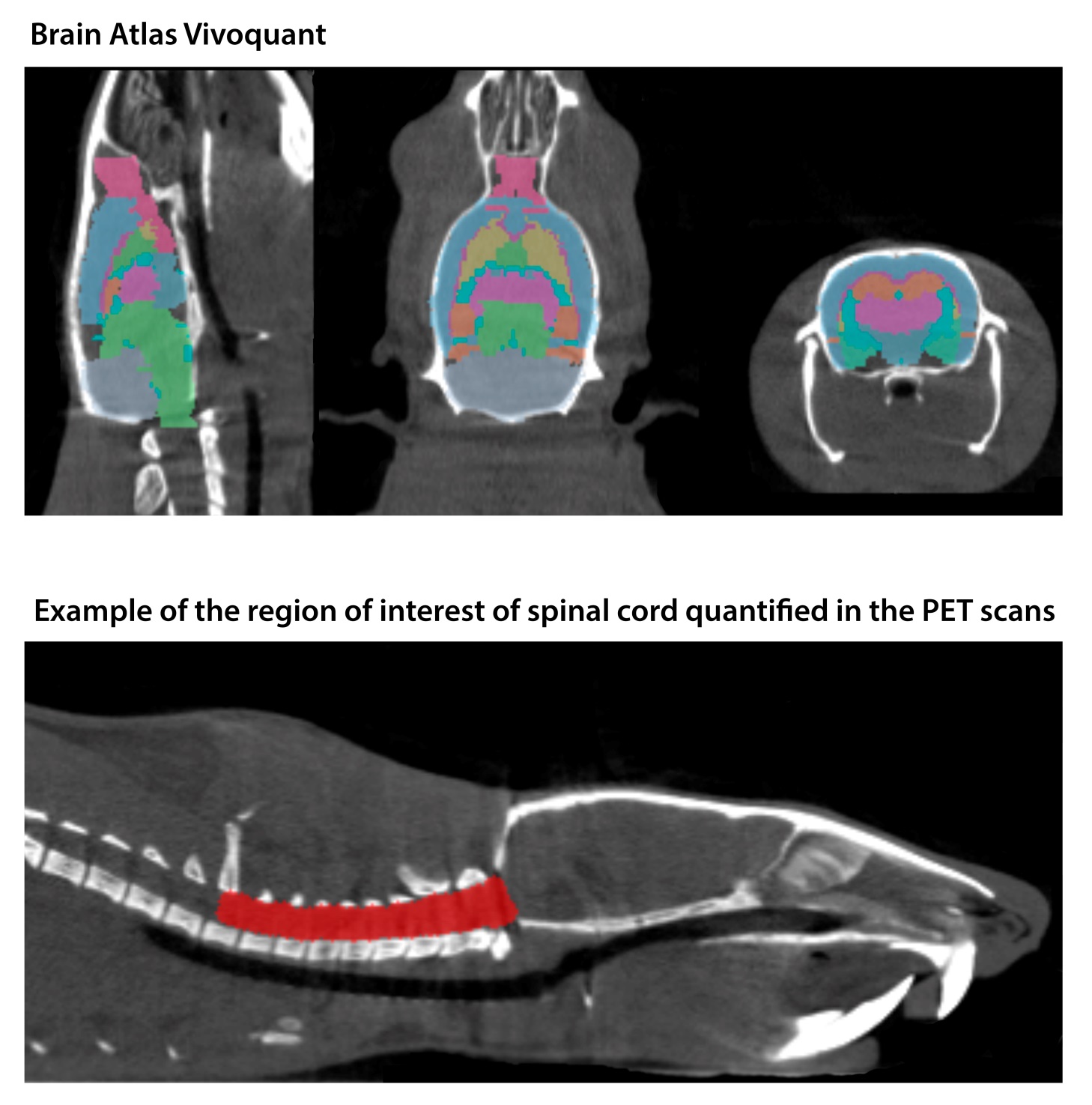


**Supplementary Figure 5.** Figure showing the region of interest drawn for the PET scans analysis. Vivoquant atlas was applied to delineate the brain stem and cerebellum, and manual region of interest was drawn on the spinal cord (Shown in red in the lower panel)


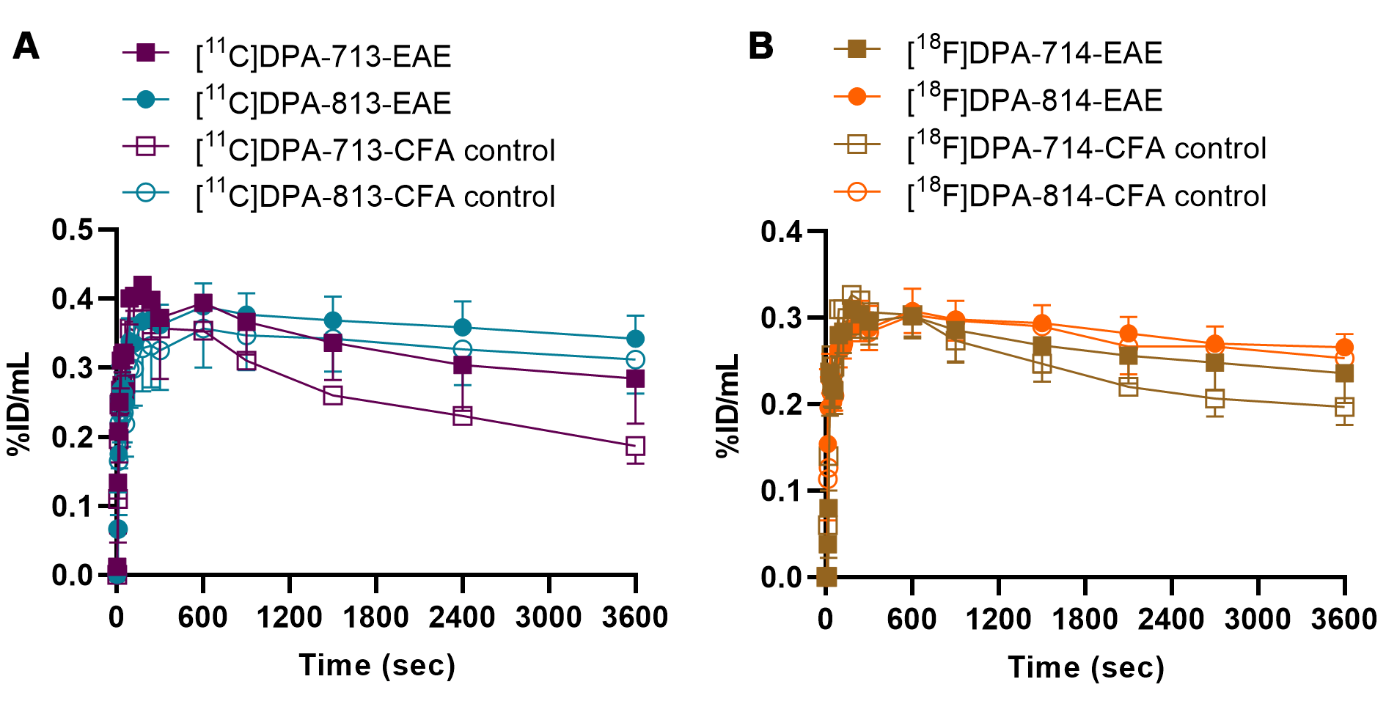


**Supplementary Figure 6.** Brain time activity curves of [^11^C]DPA-813 and [^11^C]DPA-713 **(A)**, [^18^F]DPA-814 and [^18^F]DPA-714 **(B)** in the EAE and CFA control animals. Data is expressed as percent injected dose per mL (%ID/mL).


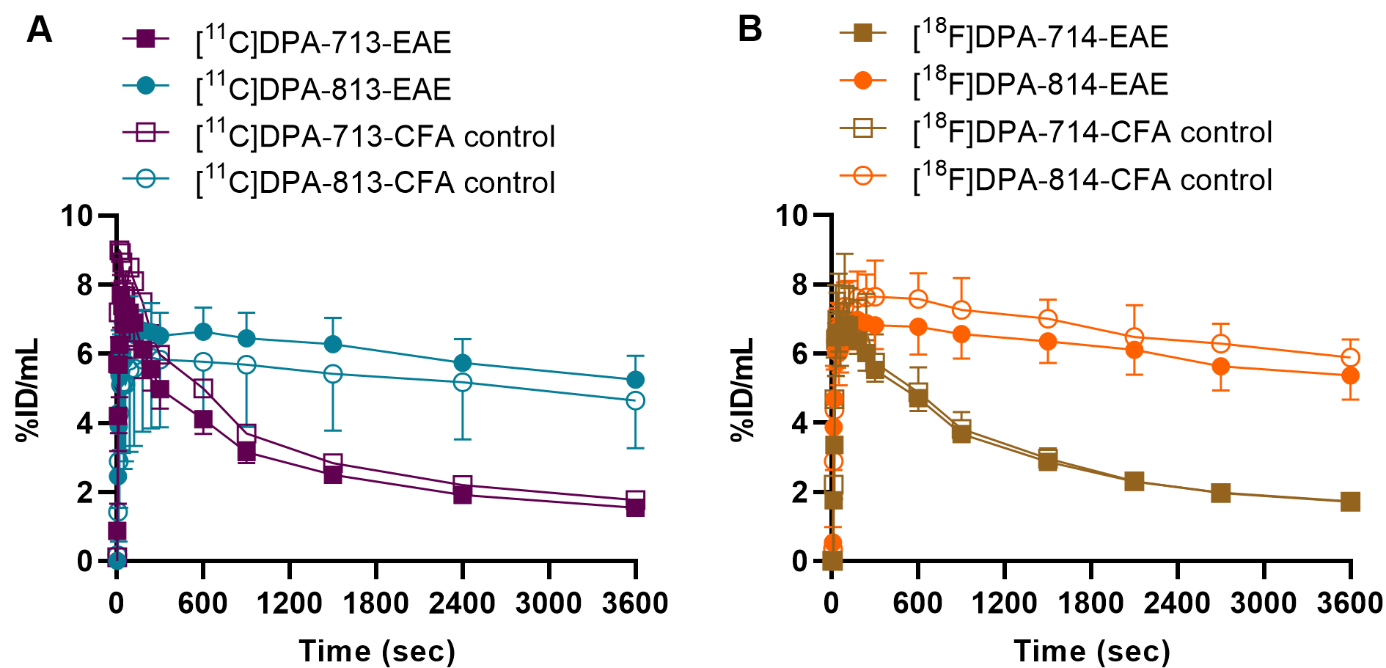


**Supplementary Figure 7.** Lungs time activity curves of [^11^C]DPA-813 and [^11^C]DPA-713 **(A)**, [^18^F]DPA-814 and [^18^F]DPA-714 **(B)** in the EAE and CFA control animals. Data is expressed as percent injected dose per mL (%ID/mL).


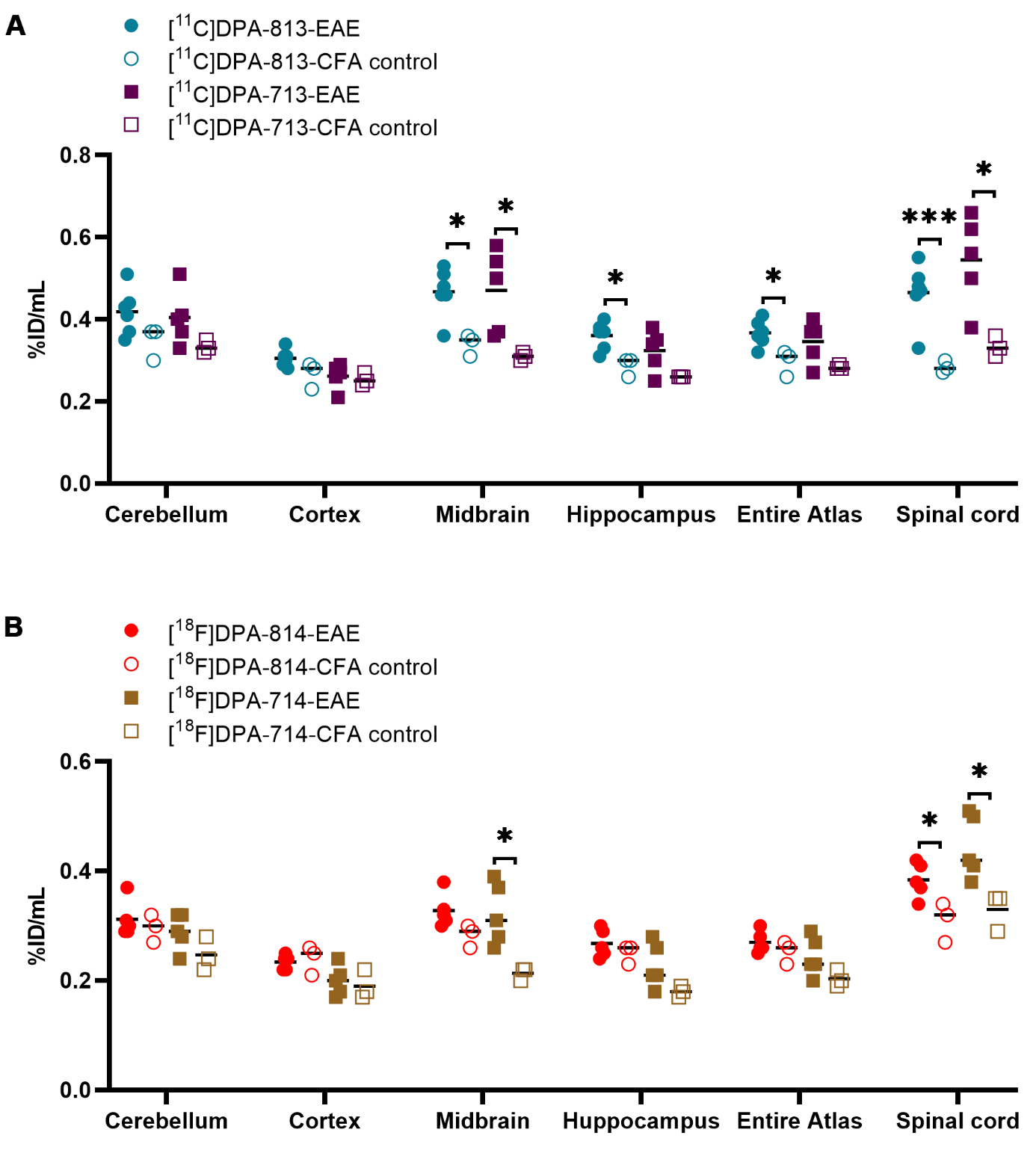


**Supplementary Figure 8.** Quantification of static PET imaging in different brain sub-regions of [^11^C]DPA-813 and [^11^C]DPA-713 **(A)**, [^18^F]DPA-814 and [^18^F]DPA-714 **(B)** in the EAE and CFA control animals. Data is expressed as percent injected dose per mL (%ID/mL). ** p<0.05; *** p<0.001*.


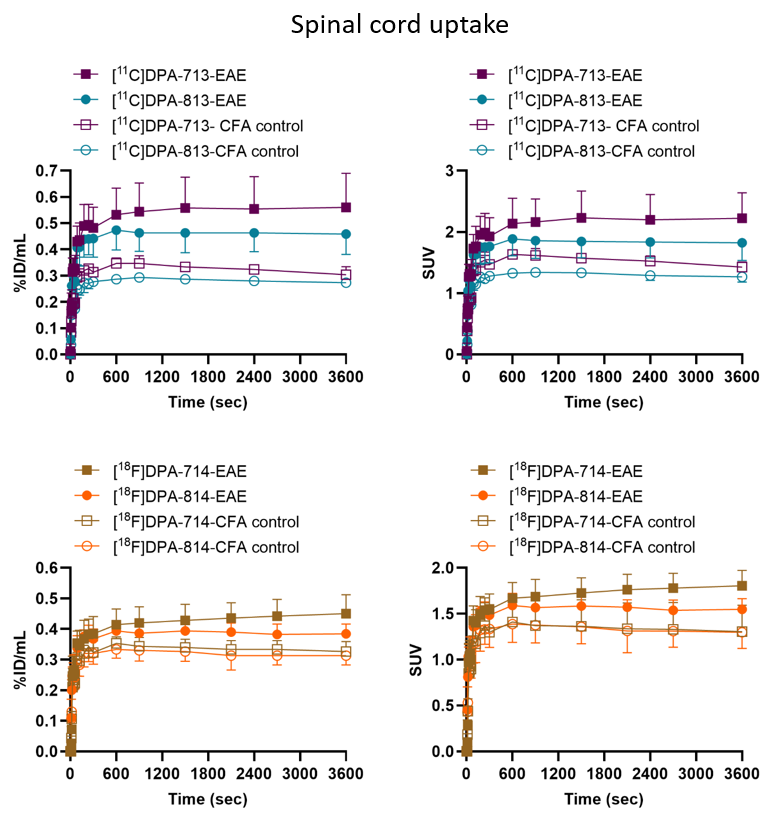

**Supplementary Figure 9.** Time activity curves (TAC) of the uptake in the spinal cord presented in %ID/mL (left) vs SUV (right) of [^11^C]DPA-813, [^11^C]DPA-713 (upper two graphs), and [^18^F]DPA-814, and [^18^F]DPA-714 (lower two graphs).

# Ex vivo validation of microglia activation in the spinal cord


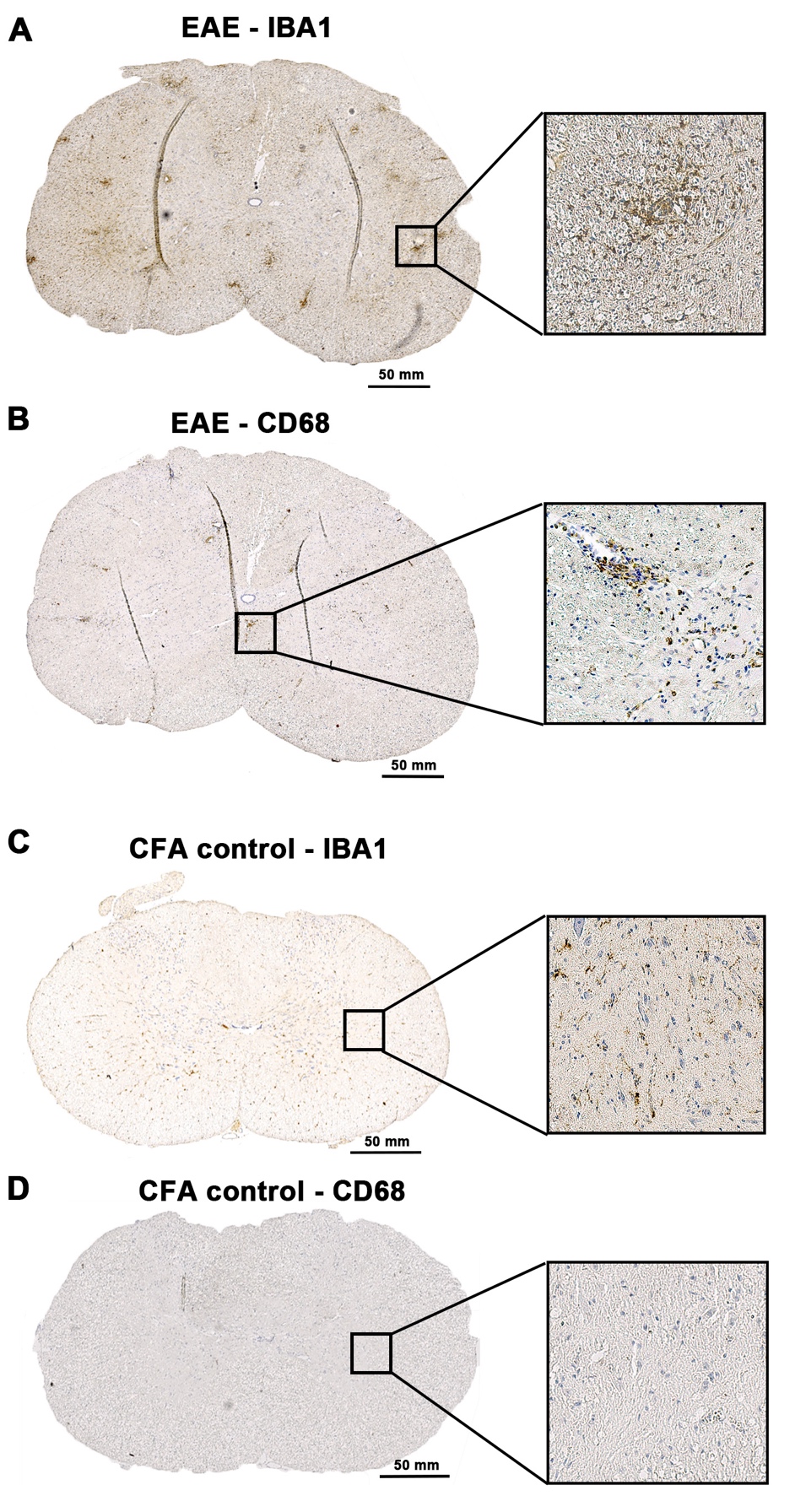


**Supplementary Figure 10.** Staining for activated microglia in the spinal cord of the EAE and CFA rats post-PET imaging. IBA1 staining in EAE **(A)** and CFA control **(C)**. CD68 staining in EAE **(B)** and CFA control **(D)**.

# In vitro off-target screening

**Supplementary Table 5.** Off-target binding to common CNS targets. 10 μM single dose (n = 2) screen performed by Eurofins Panlabs Discovery. ^a^ All targets are human receptors. ^b^ Inhibition of antagonist radioligand binding. ^c^ Inhibition of agonist radioligand binding. ^d^ Enzymatic functional readout.

|  | **% Binding Inhibition at 10 μM** | |
| --- | --- | --- |
| **Off-Target Receptor ^a^** | **DPA-813** | **DPA-814** |
| 5-HT2A ^b^ | 1 | 4 |
| 5-HT2B ^c^ | 25 | 30 |
| β2 Adrenergic receptor ^b^ | -1 | 0 |
| Central Benzodiazepine Receptor ^c^ | 51 | 18 |
| CB1 ^b^ | 4 | -6 |
| CB2 ^c^ | -16 | -22 |
| D1 ^b^ | 13 | 24 |
| D2L ^b^ | 10 | 11 |
| DAT ^b^ | 10 | 7 |
| M1 ^b^ | -13 | -14 |
| MAO-B ^d^ | 10 | 47 |
| MOP ^c^ | 15 | 24 |
| nAChR (α4/β2) ^b^ | 4 | 1 |
| NET ^b^ | 3 | 4 |
| SERT ^b^ | 4 | 2 |

# General Synthesis Experimental

Unless otherwise stated, all reactions were performed under an atmosphere of nitrogen and all solvents and reagents were used as purchased from commercial sources. Anhydrous solvents dichloromethane, tetrahydrofuran and dimethylformamide were obtained from a PureSolv MD 7 solvent purification system (Innovative Technology, Inc.). Analytical thin-layer chromatography (TLC) was performed using Merck aluminium backed silica gel 60 F254 (0.2 mm) plates and visualised under shortwave (254 nm) and/or longwave (365 nm) ultraviolet light as well as with potassium permanganate, bromocresol green and vanillin stains. Flash column chromatography was performed using Merck silica gel pore size 60 Å, 40-63 µm, 230-400 mesh with eluent mixtures reported as volume to volume (v/v) ratios or percentages. Nuclear magnetic resonance spectra were recorded using a Bruker AVANCE DRX300 (300 MHz) spectrometer at 300 K. ^1^H chemical shifts are expressed as parts per million (ppm) with residual chloroform (δ 7.26) as reference and are reported as chemical shift (δ); relative integral; multiplicity (s = singlet, d = doublet, dd = doublet of doublets, dt = doublet of triplets, t = triplet, q = quartet, m = multiplet etc.); coupling constants (*J*) reported in Hz. ^13^C chemical shifts are expressed as parts per million (ppm) with residual chloroform (δ 77.16) as a reference and reported as chemical shift (δ). Low-resolution mass spectra (LRMS) were recorded using electrospray ionisation (ESI) recorded on a Bruker AmaZon SL ion trap spectrometer. High resolution mass spectrometry (HRMS) was performed on a Bruker Apex Qe 7T Fourier Transform Ion Cyclotron Resonance mass spectrometer equipped with an Apollo II ESI/MALDI dual source. Samples were run with syringe infusion at 150 μL/h on a Cole Palmer syringe pump into the ESI source. High performance liquid chromatography (HPLC) analysis of organic purity was conducted on a Waters Alliance 2695 instrument using a SunFireTM C18 column (5 μm, 2.1 x 150 mm) and detected using a Waters 2996 photodiode array (PDA) detector set at 254 nm. Separation was achieved using water (solvent A) and acetonitrile (solvent B) at a flow rate of 0.2 mL/min with a gradient of 0% B to 100% B over 30 minutes. HPLC data are reported as percentage purity and retention time (RT) in minutes.

# Synthesis of DPA-813 and DPA-814 standards and radiolabelling precursors

**Supplementary Figure 10** Synthesis of DPA-813 and DPA-814 and radiolabelling precursors for synthesis of [^11^C]DPA-813 and [^18^F]DPA-814.

Methyl 4-isopropoxybenzoate (20.0 g, 102.9 mmol, 1.0 eq) was dissolved in anhydrous tetrahydrofuran (400 mL) with stirring at room temperature. Potassium *tert*-butoxide (25.4 g, 257.4 mmol, 2.2 eq) was added in portions as a solid [*note: reaction mixture turned from clear, colourless solution to thick, slow-stirring yellow suspension*], followed immediately by addition of acetonitrile (26.9 mL, 514.9 mmol, 5.0 eq). The reaction mixture was stirred at room temperature for 16 hours, then diluted with water (150 mL) and washed with methylene chloride (2 x 250 mL). The aqueous layer was subsequently acidified to pH < 3 using 32% aqueous hydrochloric acid and extracted with ethyl acetate (3 x 250 mL). The combined organic layers were dried (MgSO_4_) and concentrated under reduced pressure to afford a crude brown crystalline residue. Recrystallisation from 1:1 isopropanol/hexane yielded **S1** (8.5 g, 41%) as pale brown crystals. Characterisation data matched those reported previously.[1] **^1^H NMR** (300 MHz, CDCl_3_) δ 7.87 (d, *J* = 8.9 Hz, 2H), 6.93 (d, *J* = 8.9 Hz, 2H), 4.67 (hept, *J* = 6.1 Hz, 1H), 4.00 (s, 2H), 1.37 (d, *J* = 6.1 Hz, 6H); **^13^C NMR** (75 MHz, CDCl_3_) δ 185.36, 163.34, 131.00, 126.78, 115.57, 114.19, 77.25, 70.52, 29.00, 21.85; **LRMS (+ESI)** 204.14 (30 %, [M+H]^+^), 226.14 (100 %, [M+Na]^+^), 429.11 (51 %, [2M+Na]^+^); **HRMS (+ESI)** Calc. for C_12_H_13_NNaO_2_ [M+Na]^+^: 226.08385, found 226.08358.

**S1** (7.9 g, 39.0 mmol, 1.0 eq) was dissolved in anhydrous THF (200 mL) and cooled on an ice bath to 0ºC, before the addition of sodium hydride (60% w/w dispersion in mineral oil, 1.6 g, 41.0 mmol, 1.05 eq) in four portions. The resulting reaction mixture was left to stir at 0ºC for 30 minutes. 2-bromo-*N*-methyl-*N*-phenylacetamide[2] (8.9 g, 39 mmol, 1.0 eq) was subsequently added as a solid, and the reaction was left to stir with warming to room temperature for 16 hours [*note: reaction mixture turned from pale yellow suspension to pale brown slurry*]. The reaction mixture was quenched carefully with 1M aqueous hydrochloric acid (300 mL) and extracted with ethyl acetate (3 x 100 mL). The combined organics layers were washed sequentially with saturated aqueous sodium bicarbonate (100 mL) and brine (100 mL), dried (MgSO_4_), and concentrated under reduced pressure to yield crude **S2** as an orange syrup which solidified on standing and was deemed of sufficient purity to carry through to subsequent steps without further purification. **^1^H NMR** (300 MHz, CDCl_3_) δ 8.00 (d, *J* = 8.9 Hz, 2H), 7.54 – 7.37 (m, 2H), 7.33 – 7.09 (m, 3H), 6.94 (d, *J* = 9.0 Hz, 2H), 4.92 (dd, *J* = 9.2, 4.7 Hz, 1H), 4.66 (hept, *J* = 6.1 Hz, 1H), 3.26 (s, 3H), 3.05 (dd, *J* = 16.5, 9.1 Hz, 1H), 2.58 (dd, *J* = 16.5, 4.7 Hz, 1H), 1.37 (d, *J* = 6.1 Hz, 6H); **^13^C NMR** (75 MHz, CDCl_3_) δ 187.47, 168.34, 163.27, 142.91, 131.53, 130.20, 128.53, 127.36, 126.48, 117.41, 115.52, 70.41, 37.54, 33.97, 33.52, 21.87; **LRMS (+ESI)** 373 (100%, [M+Na]^+^); **HRMS (+ESI)** Calc. for C_21_H_22_N_2_NaO_3_ [M+Na]^+^: 373.15226, found 373.15261.

To a solution of **S2** (13.6 g, 39.0 mmol, 1.0 eq) in absolute ethanol (140 mL) was added hydrazine hydrate (3.8 mL, 78.0 mmol, 2.0 eq) followed by glacial acetic acid (3.7 mL, 58.5 mmol, 1.5 eq). The reaction mixture was heated at reflux for 90 minutes, until no starting material remained by TLC analysis. Acetylacetone (6.0 mL, 58.5 mmol, 1.5 eq) was added directly to the hot reaction mixture, which was stirred at reflux for 16 hours. After cooling to room temperature, the volatiles were removed under reduced pressure and the dark brown residue redissolved in methylene chloride (250 mL), followed by washing with saturated aqueous sodium bicarbonate (50 mL), water (50 mL), and brine (50 mL). The organic layer was dried (MgSO_4_) and partially decolourised with activated charcoal before being concentrated to a pale brown oil under reduced pressure. Trituration from a refluxing mixture of 3:1 hexane/toluene afforded **S3** (9.3 g, 56% over three steps) as a fine white powder after cooling to ambient temperature. **^1^H NMR** (300 MHz, CDCl_3_) δ 7.66 (d, *J* = 8.7 Hz, 2H), 7.45 – 7.25 (m, 5H), 6.97 (d, *J* = 8.8 Hz, 2H), 6.47 (s, 1H), 4.62 (hept, *J* = 6.1 Hz, 1H), 3.71 (s, 2H), 3.29 (s, 3H), 2.73 – 2.46 (m, 6H), 1.37 (d, *J* = 6.0 Hz, 6H); **^13^C NMR** (75 MHz, CDCl_3_) δ 171.15, 158.12, 157.40, 154.88, 147.73, 144.62, 144.25, 129.82, 129.63, 127.53, 126.12, 115.97, 108.12, 100.74, 69.94, 37.78, 29.27, 24.67, 22.07, 16.89; **LRMS (+ESI)** 429.18 (100 %, [M+H]^+^), 451.20 (67 %, [M+Na]^+^); **HRMS (+ESI)** Calc. for C_26_H_29_N_4_O_2_ [M+H]^+^: 429.22850, found 429.22874; **IR (*ν*_max_/cm^−1^)** 1651, 1611, 1561, 1480, 1439, 1377, 1302, 1240, 1183, 1116, 950, 838, 777, 703, 640, 628, 563, 522, 409; **HPLC** 97.95% (λ_max_ = 254 nm), RT: 26.539.

**1** was synthesised using a modified literature procedure.[3] To **S3** (433 mg, 1.0 mmol, 1.0 eq) was added methanesulfonic acid (6.5 mL) at room temperature. The resulting solution was stirred at the same temperature for 30 minutes before basifying to pH ~8 with 28% aqueous ammonia and cooling to 0ºC. The resultant precipitate was isolated by vacuum filtration, rinsed sparingly with ice-cold water, and dried under high vacuum to yield **1** (357 mg, 92%) as an off-white powder. **^1^H NMR** (300 MHz, CDCl_3_) δ 7.72 (s, 1H), 7.53 (d, J = 8.2 Hz, 2H), 7.41 – 7.26 (m, 5H), 6.86 – 6.75 (m, 2H), 6.45 (s, 1H), 3.74 (s, 2H), 3.25 (s, 3H), 2.69 (s, 3H), 2.53 (s, 3H); **^13^C NMR** (75 MHz, CDCl_3_) δ 171.7, 157.6, 157.0, 155.2, 147.6, 144.8, 143.9, 129.8, 129.7, 127.6, 127.4, 125.2, 115.7, 108.2, 100.4, 37.9, 29.4, 24.5, 16.9; **LRMS (+ESI)** *m/z*: 387 ([M+H]^+^, 80%), 409 ([M+Na]^+^, 100%); **HRMS** Calc. for C_23_H_22_N_4_NaO_2_ [M+Na]^+^: 409.16350, found 409.16360**; IR (*ν*_max_/cm^−1^)** 3233, 1632, 1586, 1528, 1486, 1432, 1390, 1271, 1231, 1165, 1114, 839, 769, 702, 639, 565, 521, 414; **HPLC** 97.13% (λ_max_ = 254 nm), RT: 20.63.

To a solution of **1** (50 mg, 0.13 mmol, 1 eq) in DMF (2 mL) was added C_2_CO_3_ (211.8 mg, 0.65 mmol, 5 eq) and MeI (20.24 uL, 0.325 mmol, 2.5 eq). The reaction mixture was warmed to 50 ºC and stirred for 4 days. The crude product was precipitated out with deionised water (20 mL) and centrifuged at 722 rpm at 4 ºC for 25 mins. Purification of the crude product by flash column chromatography on silica gel using methanol/dichloromethane (2% v/v) followed by recrystallisation from isopropanol/hexane afforded **DPA-813** (11.4 mg, 0.028 mmol, 22%) as a white powder. **mp** 158.8-162.0 °C; **^1^H NMR** (500 MHz, CDCl_3_) δ 7.68 (d, J = 8.6 Hz, 2H), 7.41 – 7.36 (m, 2H), 7.34 – 7.27 (m, 3H), 7.02 – 6.96 (m, 2H), 6.49 (s, 1H), 3.87 (s, 3H), 3.73 (s, 2H), 3.28 (s, 3H), 2.72 (s, 3H), 2.56 (s, 3H); **^13^C NMR** (126 MHz, CDCl_3_) δ 171.12, 159.79, 157.43, 154.80, 147.72, 144.63, 144.23, 129.82, 129.64, 127.53, 126.37, 113.99, 108.15, 100.75, 77.25, 55.36, 37.79, 29.27, 24.67, 16.89; **IR** (v_max_, cm^-1^) 3636.52, 2836.45, 1642.51, 1245.05, 835.29, 699.66, 556.32; **LRMS** (+ESI) 401 (100%, [M+H]^+^), 423 (60%, [M+Na]^+^); **HRMS** Calc. for C_24_H_25_N_4_O_2_ [M+H]^+^: 401.19720, found 401.19728; **HPLC** 97.2% (λ_max_ = 254 nm), RT: 24.05.

To **1** (200 mg, 0.517 mmol, 1 eq.) in anhydrous DMF was added sodium hydride (22.76 mg, 60% w/w dispersion in mineral oil, 1.1 eq.) at 0 ºC. The reaction mixture was warmed to ambient temperature and stirred for 1 h. 1,2-Bis(tosyloxy)ethane (229 mg, 1.2 eq.) was added and the reaction mixture left to stir at room temperature for 3 h. The reaction mixture was diluted with ethyl acetate and washed with 5% aqueous lithium chloride, then dried over anhydrous magnesium sulfate and concentrated *in vacuo*. Purification of the crude product by flash column chromatography on silica gel using methanol/dichloromethane (0.5–2% v/v) afforded **2** (122 mg, 40%) as a white powder. **^1^H NMR** (300 MHz, CDCl_3_) δ 7.88 – 7.79 (m, 2H), 7.67 (d, *J* = 8.7 Hz, 2H), 7.45 – 7.36 (m, 1H), 7.33 (dd, *J* = 12.0, 7.7 Hz, 6H), 6.93 – 6.82 (m, 2H), 6.48 (s, 1H), 4.40 (dd, *J* = 5.7, 3.8 Hz, 2H), 4.21 (dd, *J* = 5.9, 3.7 Hz, 2H), 3.70 (s, 2H), 3.28 (s, 3H), 2.70 (s, 3H), 2.54 (s, 3H), 2.45 (s, 3H). **^13^C NMR** (75 MHz, CDCl_3_) δ 171.08, 158.18, 157.51, 154.54, 147.69, 145.04, 144.62, 144.18, 132.88, 129.92, 129.85, 129.67, 128.04, 127.51, 127.14, 114.63, 108.25, 100.79, 68.13, 65.50, 37.79, 29.25, 24.67, 21.69, 16.87.; **LRMS (+ESI)** *m/z*: 607 ([M+Na]^+^, 100%), 585 (M+H]^+^, 63%); **HRMS** Calc. for C_32_H_32_N_4_NaO_5_S [M+Na]^+^: 607.19856, found 607.19920**; IR (*ν*_max_/cm^−1^)** 1651, 1441, 1363, 1238, 1175, 1111, 1019, 919, 845, 817, 775, 704, 664, 643, 575, 552, 511; **HPLC**: 98.07% (λ_max_ = 254 nm), RT: 27.12.

A solution of **1** (40 mg, 0.104 mmol, 1 eq.) and K_2_CO_3_ (72 mg, 0.518 mmol, 5 eq.) in DMF (1.7 mL) was stirred at room temperature for five minutes. To this was added 1-bromo-2-fluoroethane (300 uL, 0.156 mmol, 40 uL/mL, 1.5 equiv.) in DMF (2 mL) and the reaction mixture stirred at 40 °C for six days. The reaction mixture was diluted with ethyl acetate and the organic layer washed with 5% aqueous lithium chloride solution and water. The collected organics were then dried over anhydrous magnesium sulfate and concentrated *in vacuo*. Recrystallization from isopropyl alcohol afforded **DPA-814** (25 mg, 56%) as off-white crystals. **^1^H NMR** (300 MHz, CDCl_3_) δ 7.77 – 7.63 (m, 2H), 7.44 – 7.34 (m, 2H), 7.33 – 7.24 (m, 3H), 7.06 – 6.98 (m, 2H), 6.48 (d, J = 1.0 Hz, 1H), 4.92 – 4.64 (m, 2H), 4.37 – 4.18 (m, 2H), 3.72 (s, 2H), 3.28 (s, 3H), 2.70 (d, J = 0.9 Hz, 3H), 2.54 (s, 3H); **^13^C NMR** (75 MHz, CDCl_3_) δ 171.20, 158.75, 157.56, 154.76, 147.83, 144.74, 144.36, 130.40, 130.04, 129.73, 127.64, 127.18, 114.85, 108.31, 100.95, 82.05 (d, *J* = 170.9 Hz), 67.34 (d, *J* = 20.4 Hz), 37.91, 29.38, 24.77, 16.97; **^19^F NMR** (471 MHz, CDCl_3_) δ -223.89; **LRMS (+ESI)** *m/z*: 433 ([M+H]^+^, 93%), 455 ([M+Na]^+^, 100%); **HRMS** Calc. for C_25_H_25_FN_4_NaO_2_ [M+Na]^+^: 455.18537, found 455.18550**; IR** 1650, 1612, 1561, 1479, 1440, 1382, 1248, 1181, 1114, 1074, 1046, 921, 900, 834, 779, 702, 639, 624, 557, 515, 418; **HPLC**: 98.83% (λ_max_ = 254 nm), RT: 24.09.

#

# NMR Spectra of novel compounds

**S2**


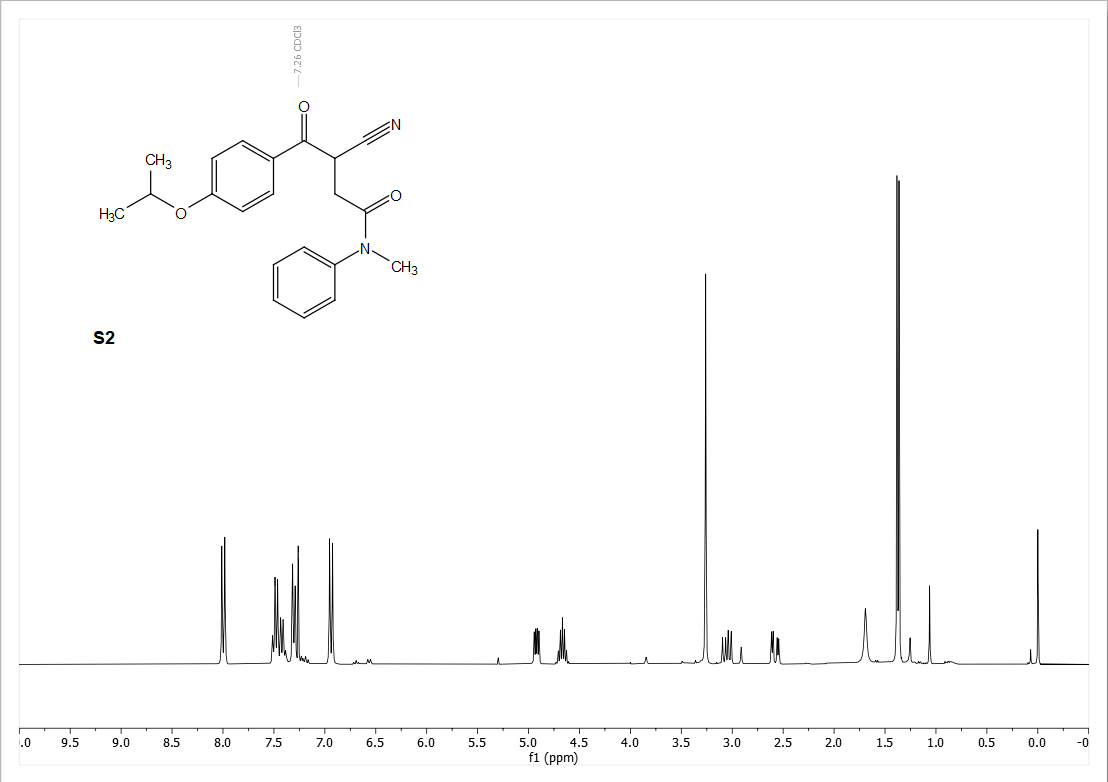


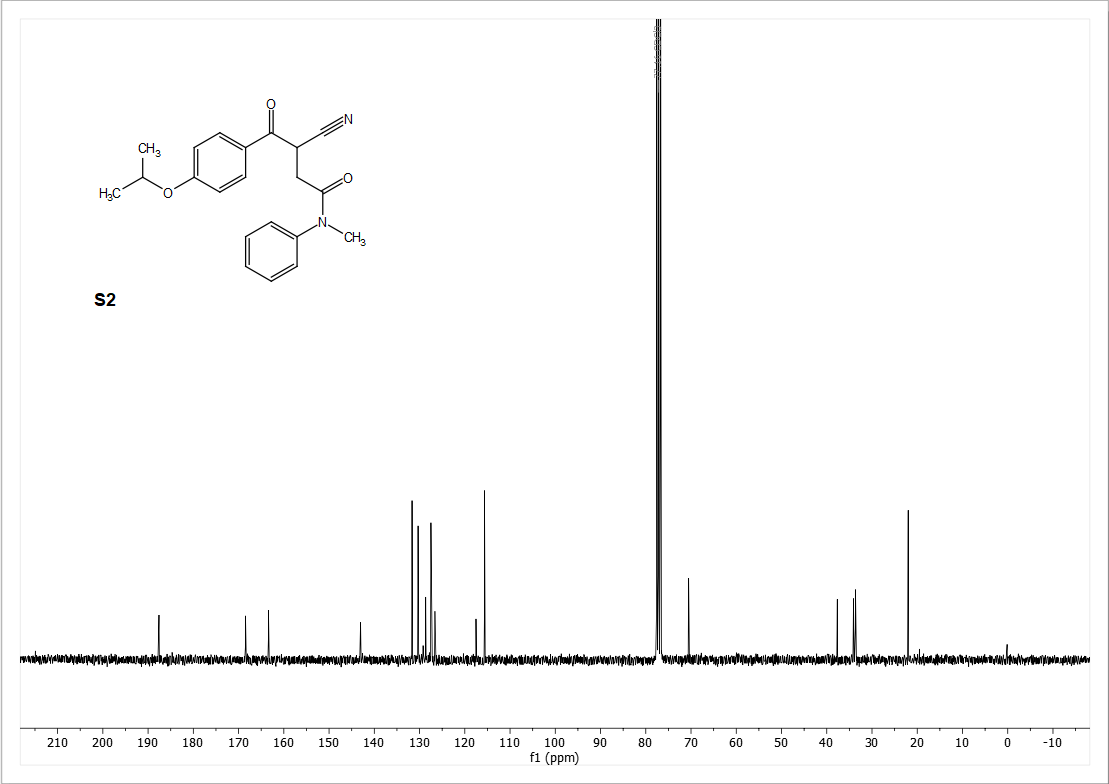


**S3**


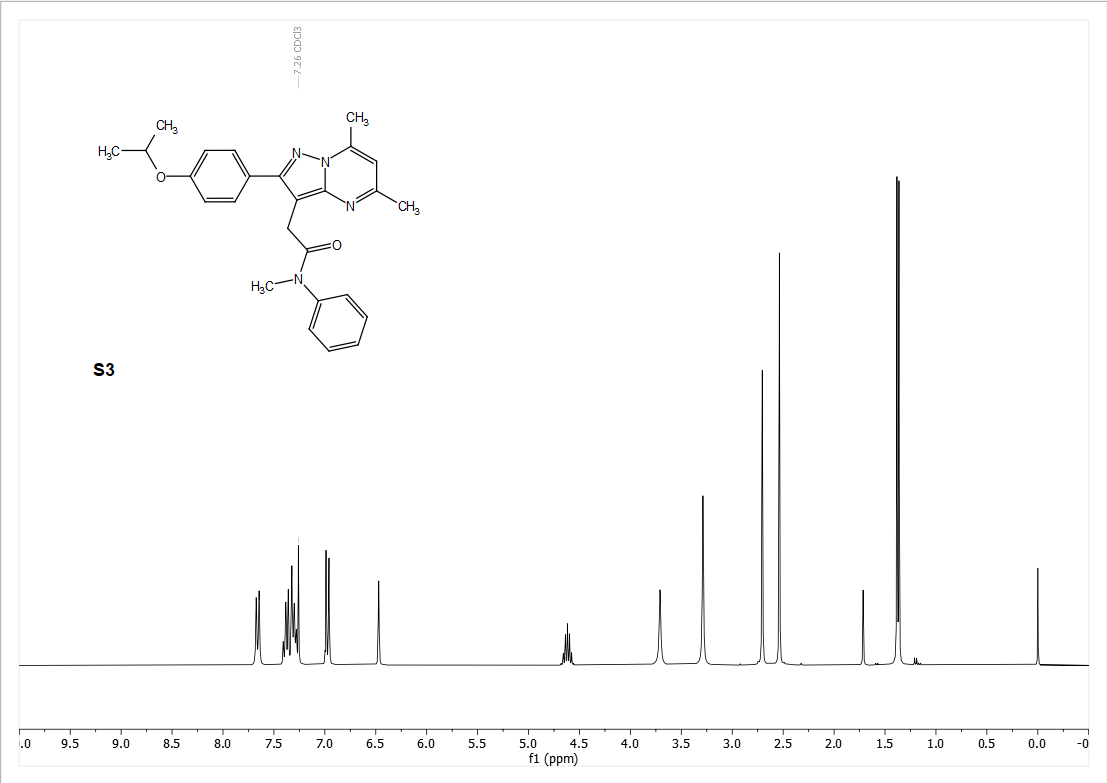


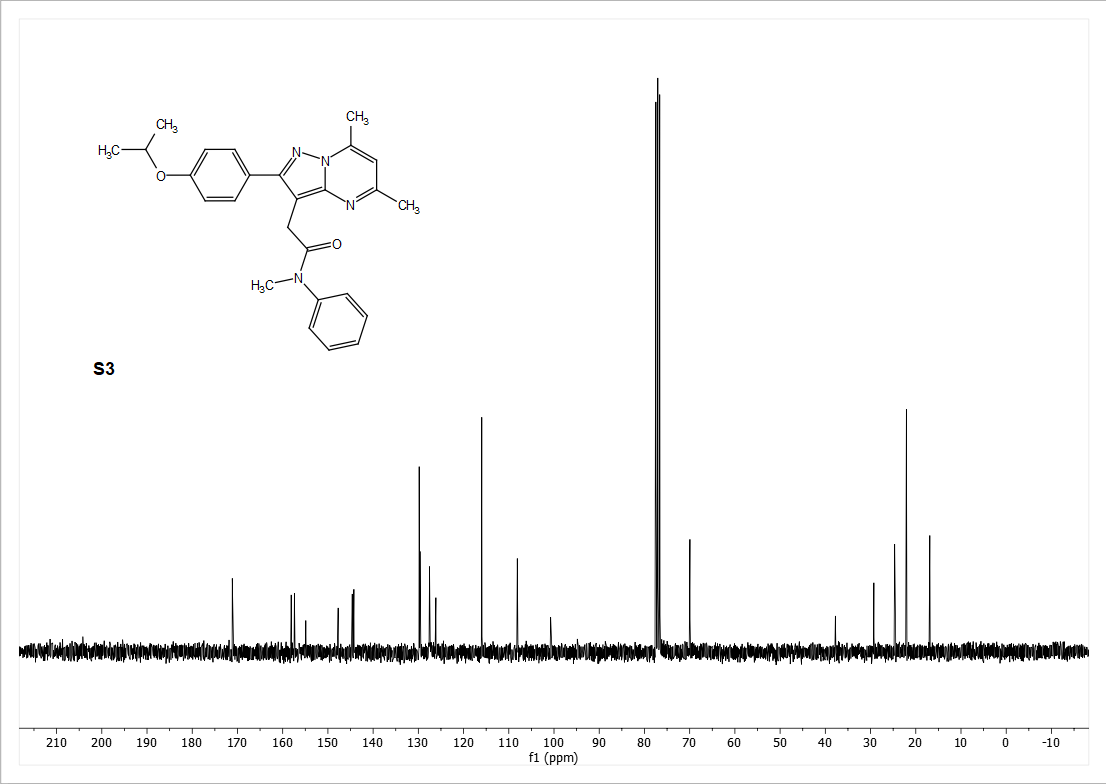


**1**


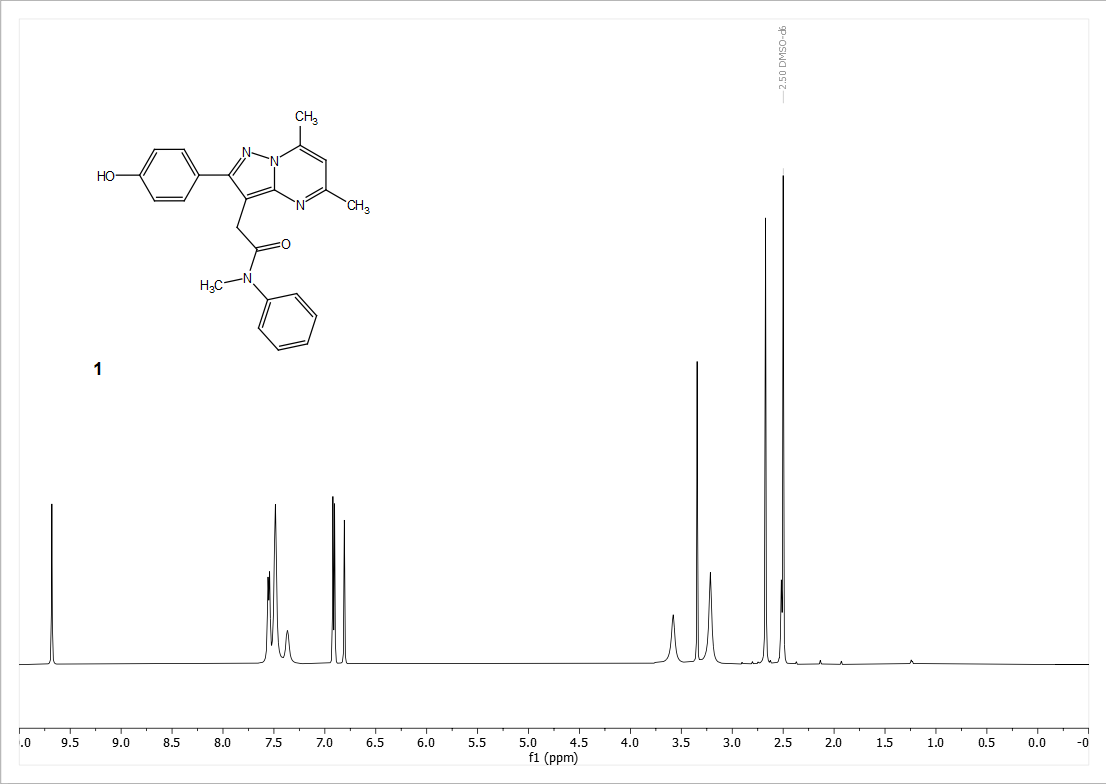


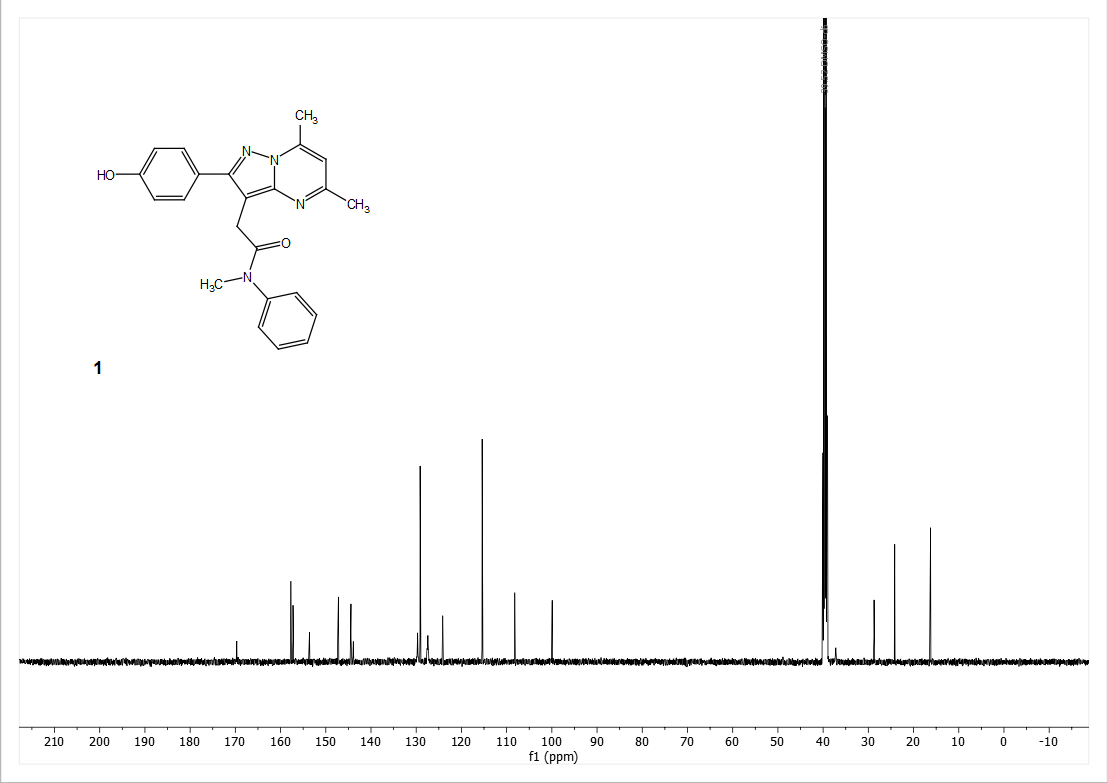


**DPA-813**


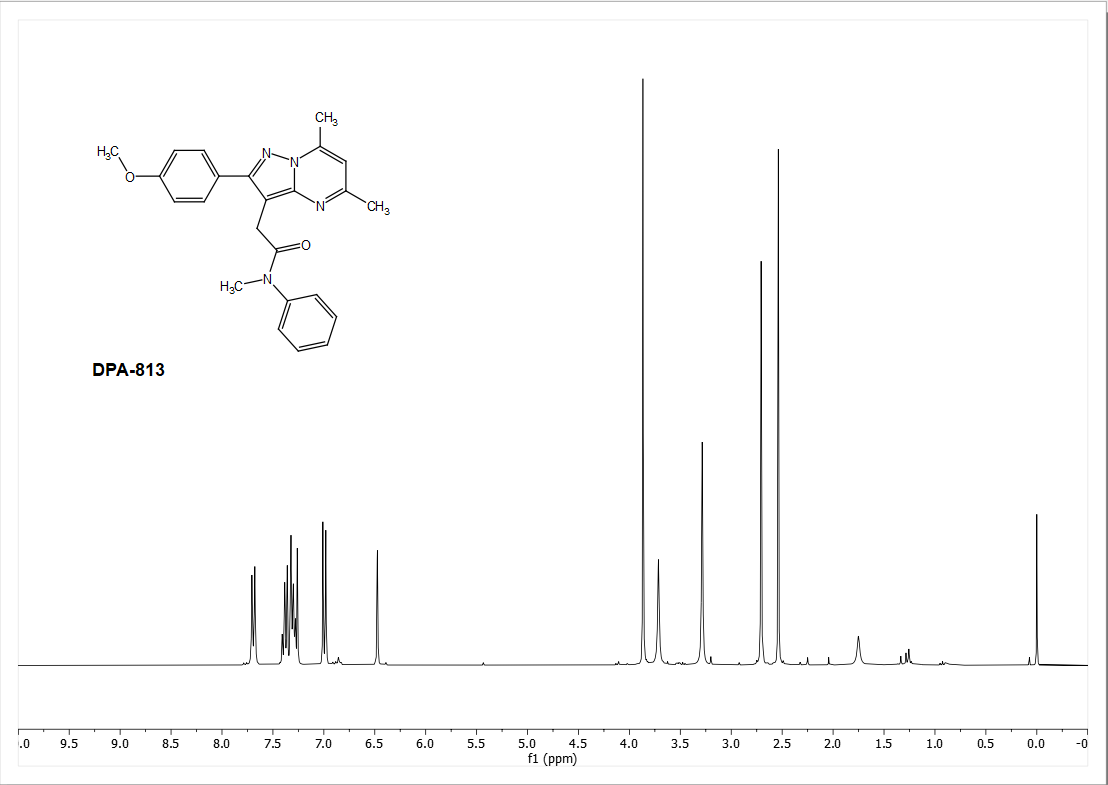


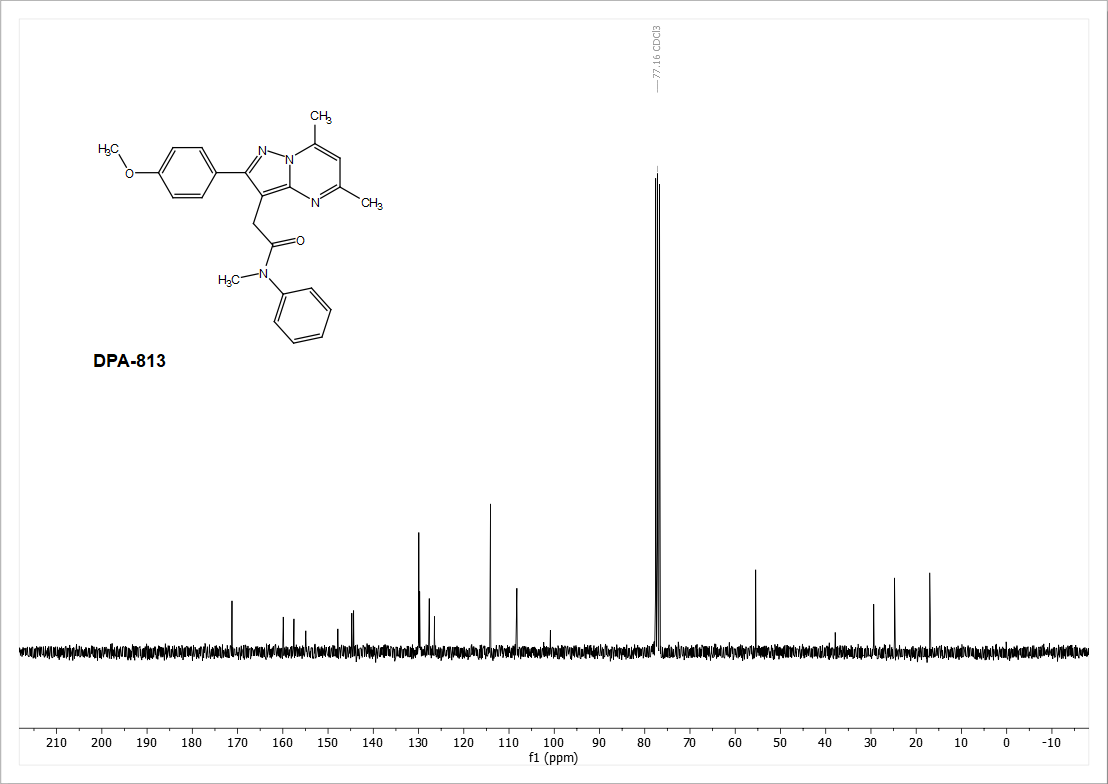


**2**


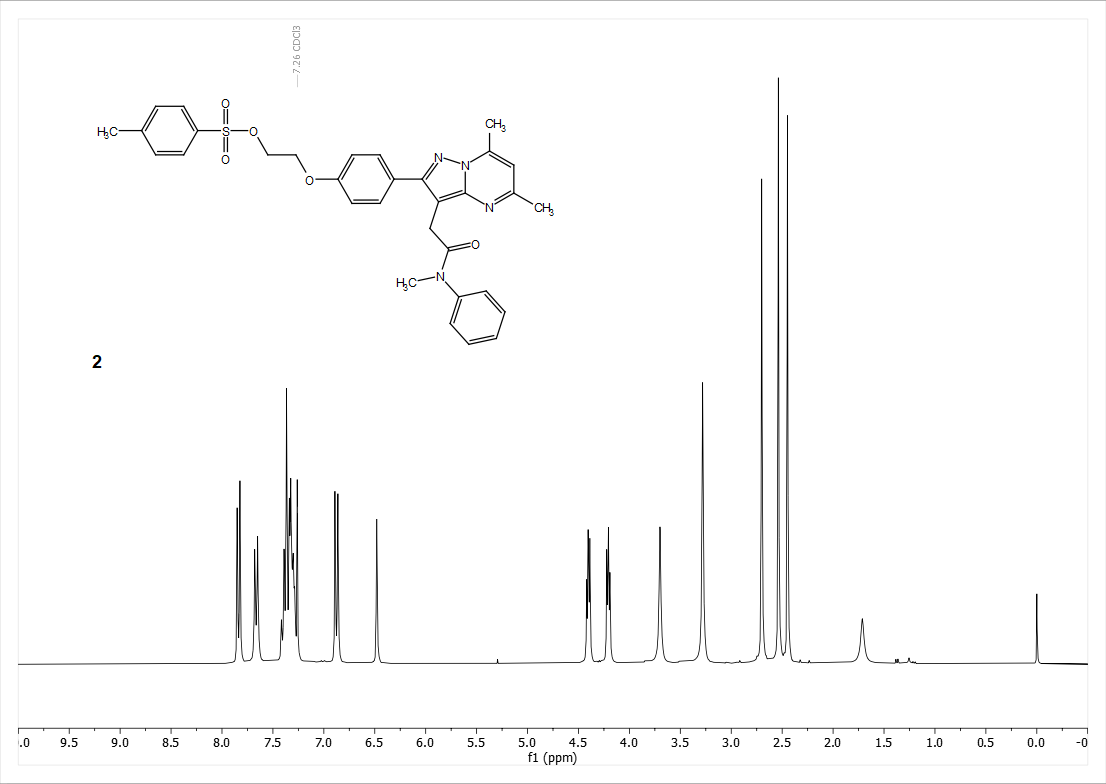


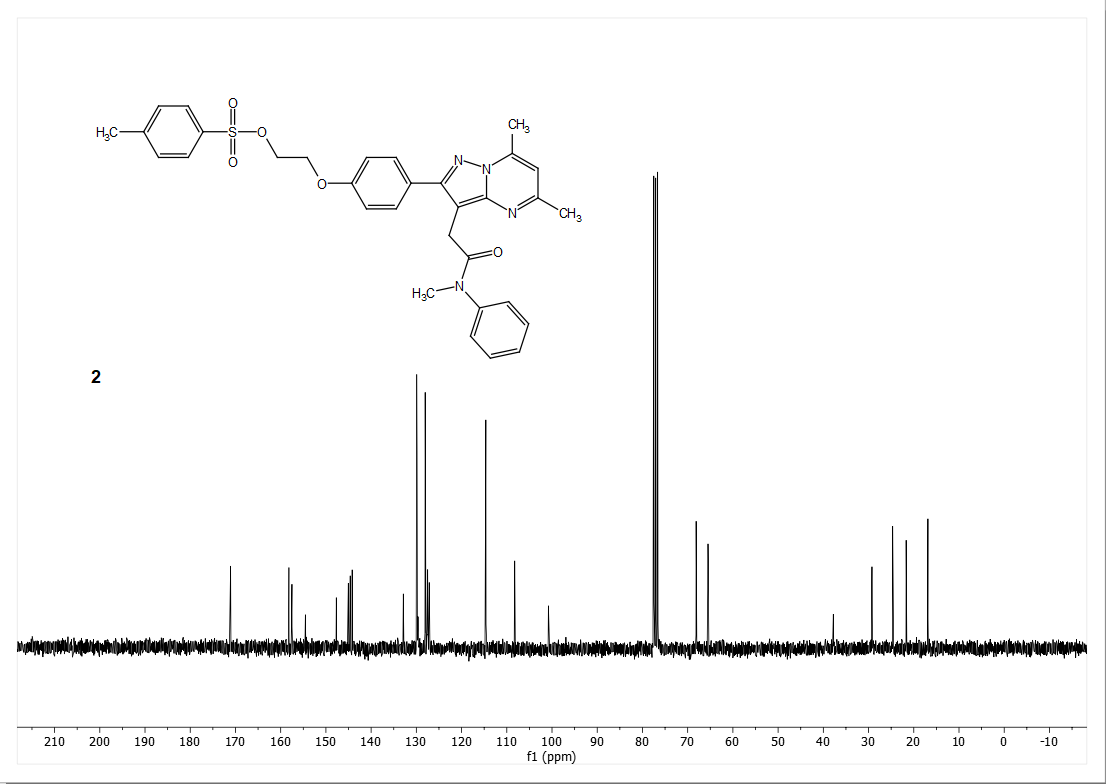


**DPA-814**


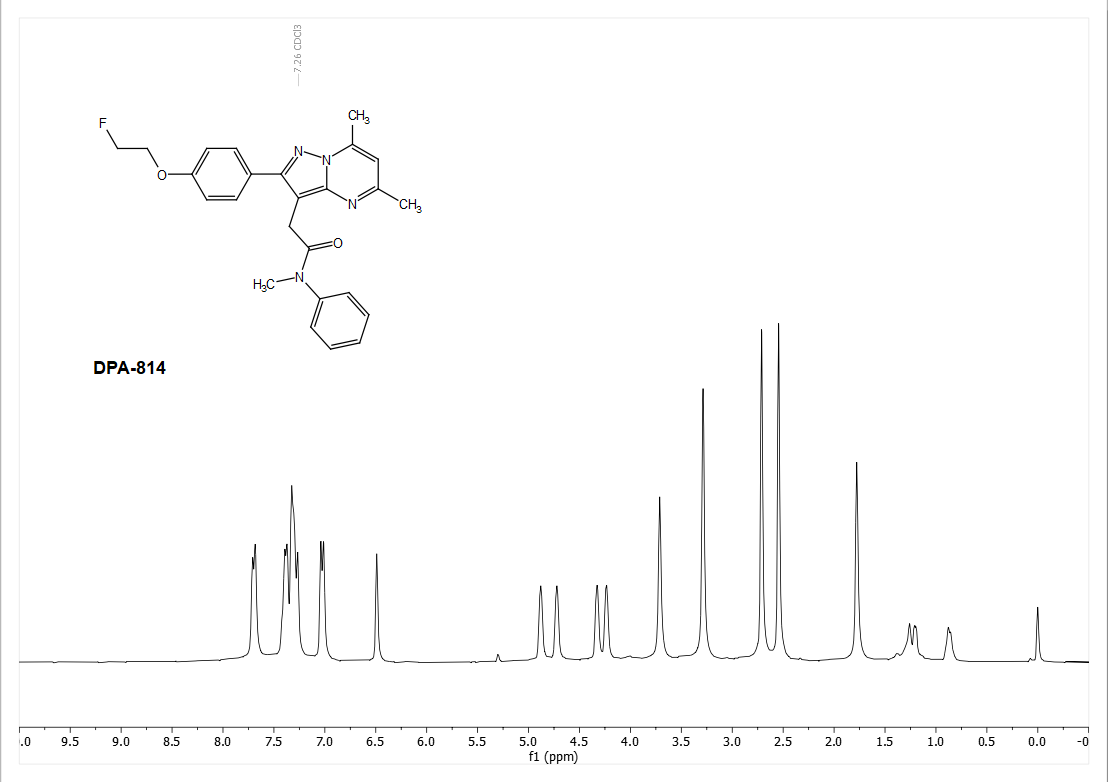


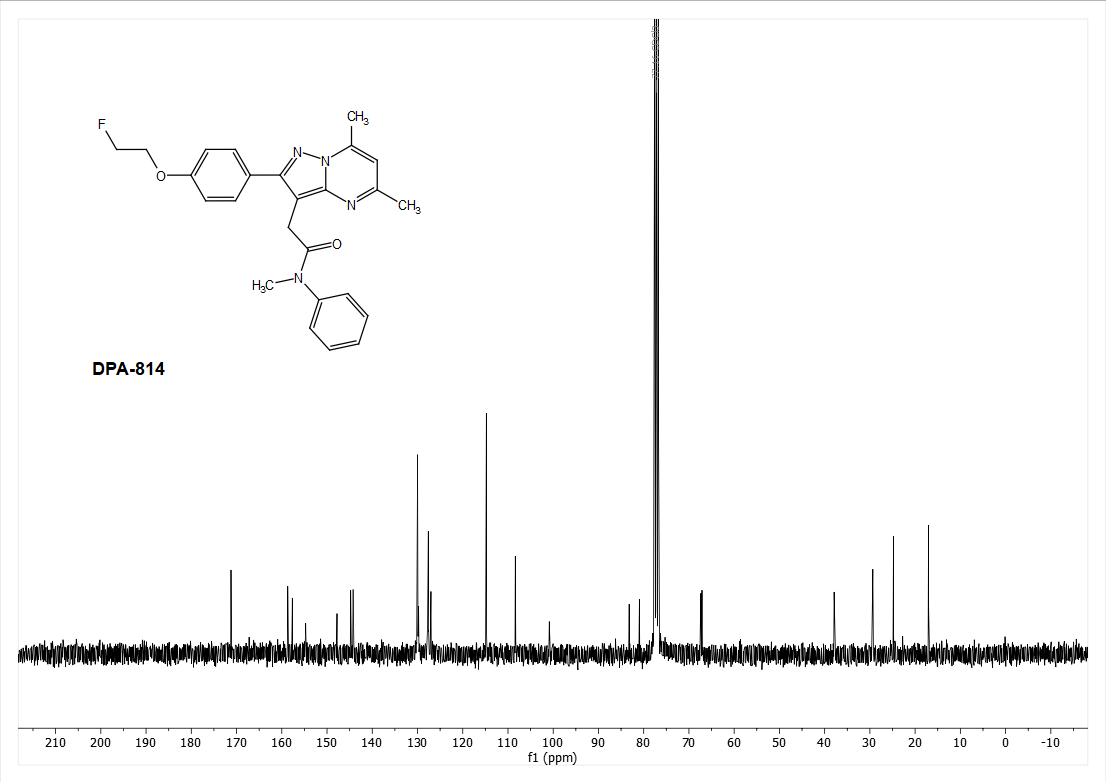


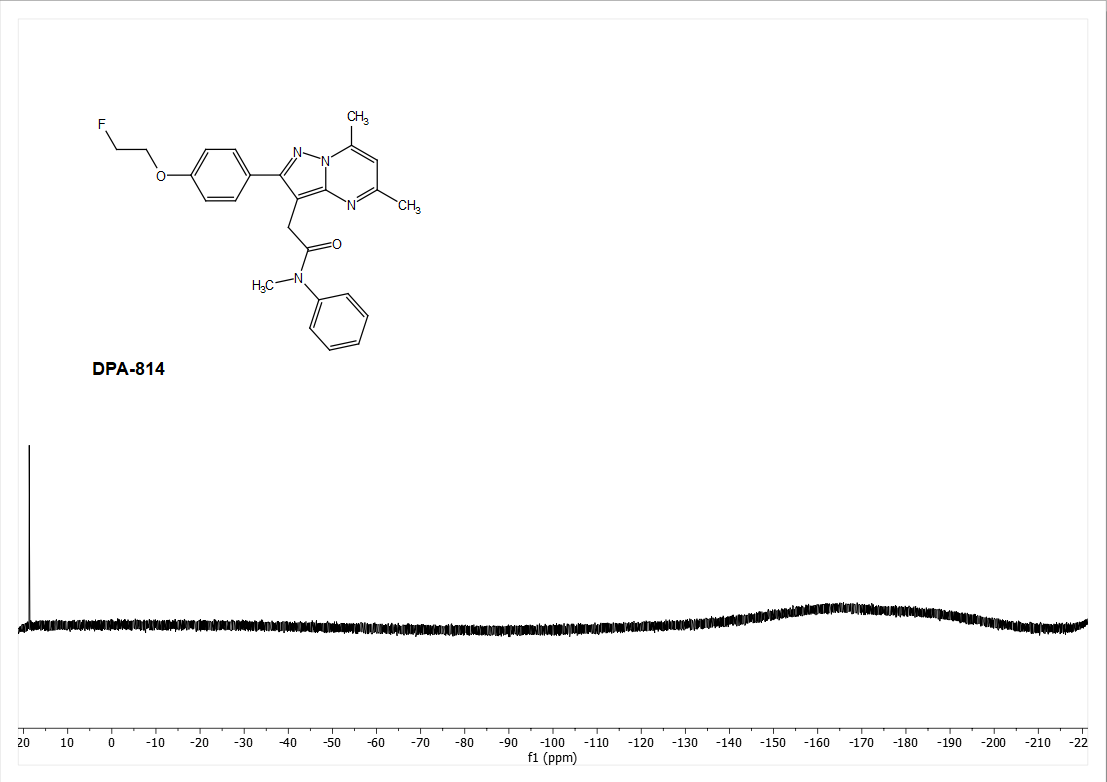


# HPLC Purity Analysis of Precursors and Standards

**1**


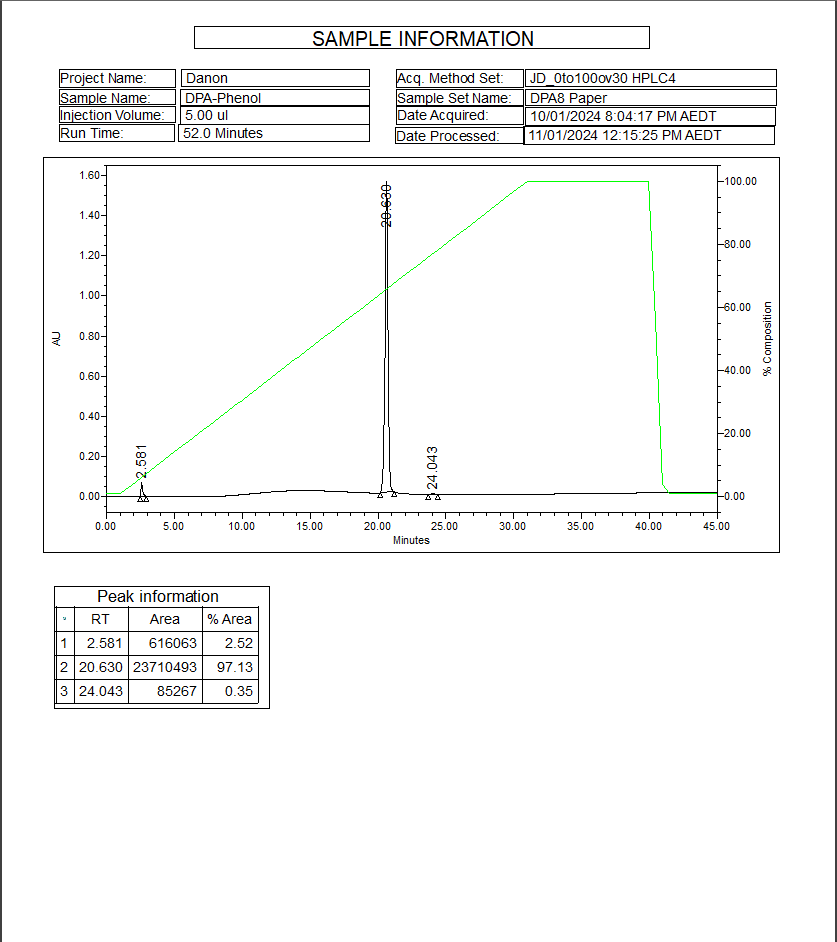


**DPA-813**


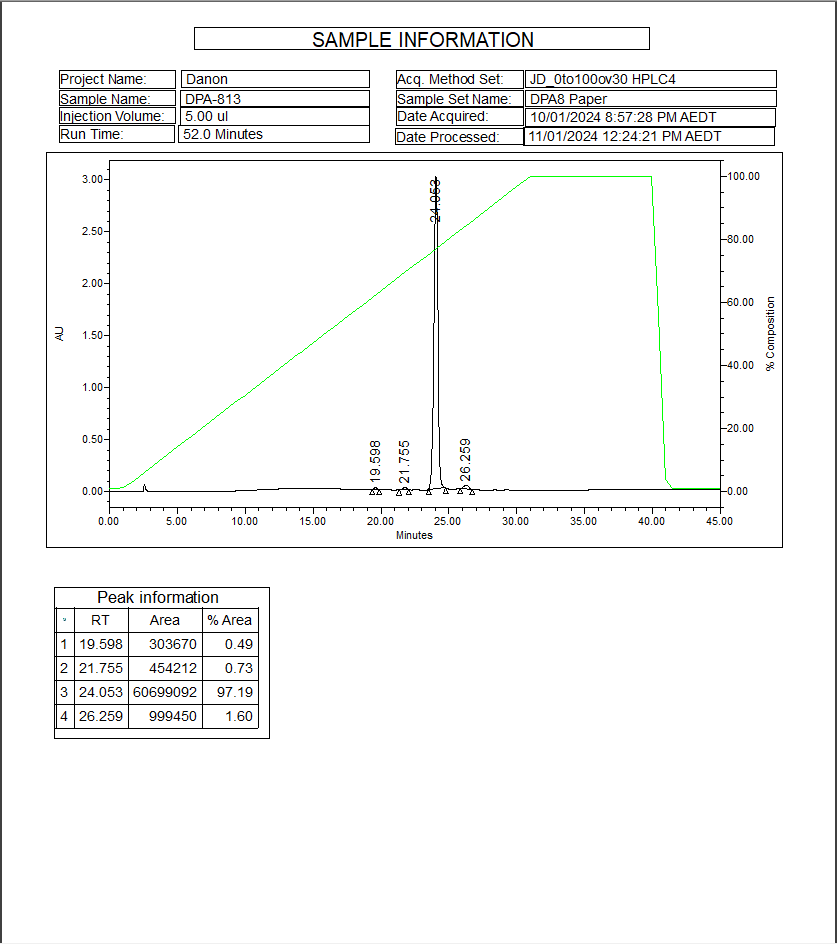


**2**


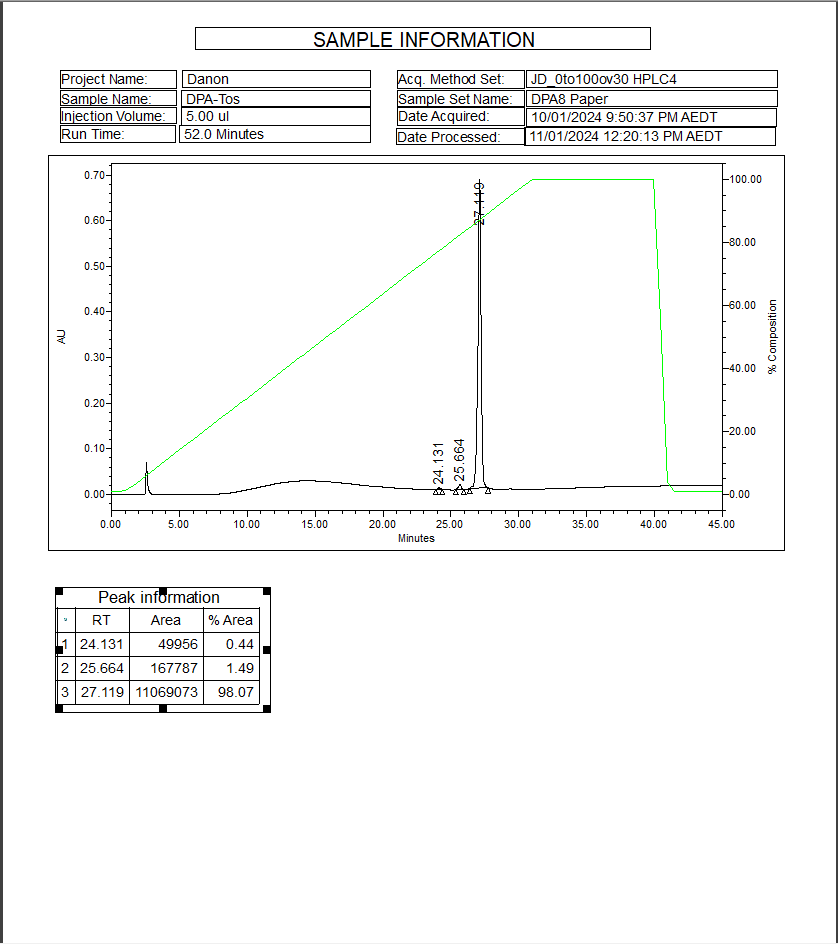


**DPA-814**


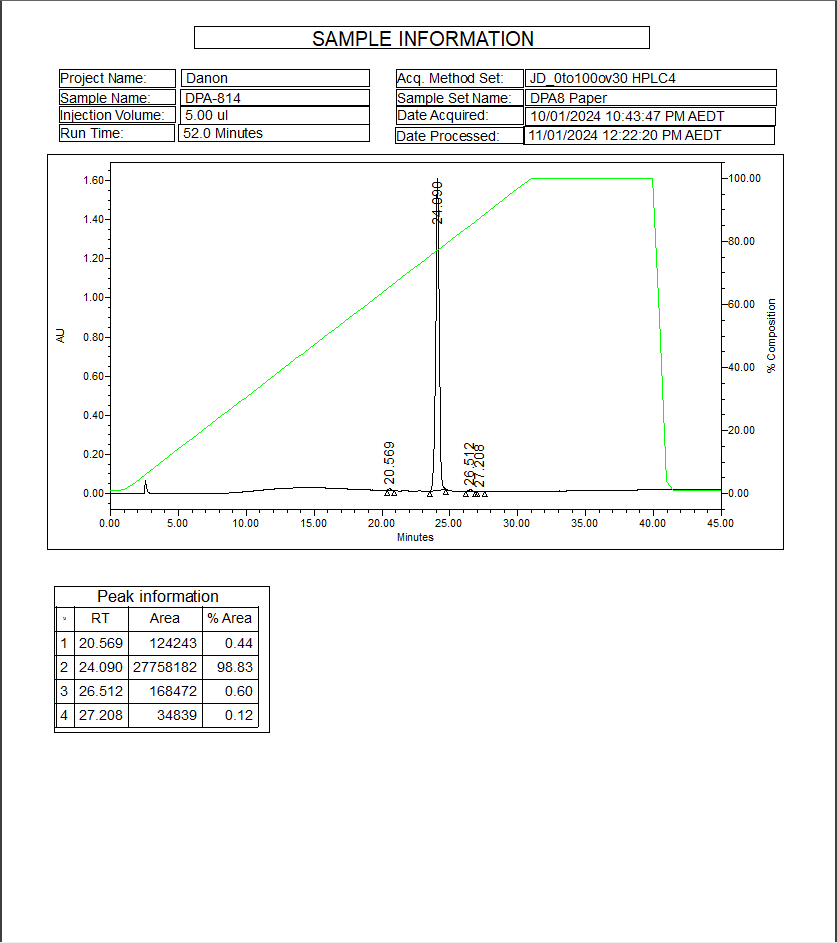


# Radioligand Binding Curves

**Supplementary Figure 11** Dose-response curves of DPA-813, DPA-814, and DPA-714 showing competitive binding to human WT (red) and A147T (blue) TSPO vs [^3^H]PK 11195. Assay details can be found in the Materials and Methods section of the manuscript.


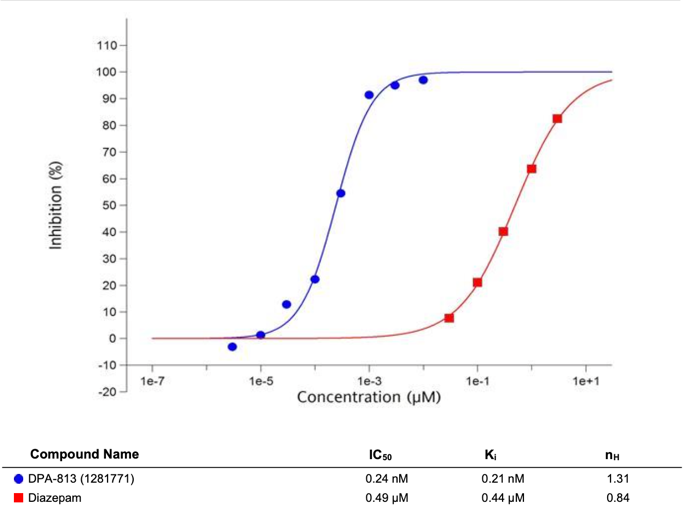

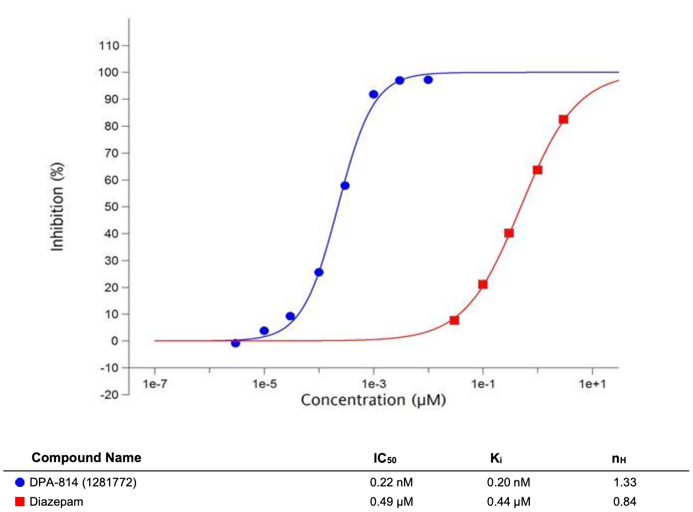


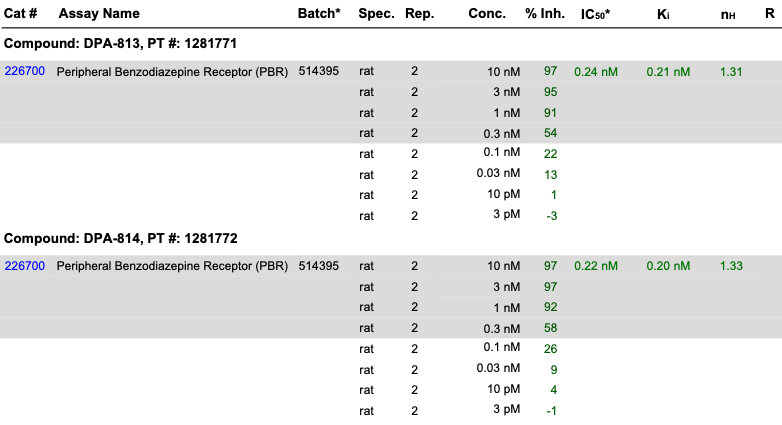


**Supplementary Figure 12** Dose-response curves and binding affinities of DPA-813 and DPA-814 showing competitive binding to rat TSPO vs [^3^H]PK 11195. Method from Eurofins: Heart of male Wistar derived rats weighing 175 ± 25 g are used to prepare peripheral benzodiazepine membrane receptors [TSPO] in modified Tris-HCl buffer pH 7.5. A 0.25 mg aliquot is incubated with 0.3 nM [^3^H]PK 11195 for 15 minutes at 25ºC. Non-specific binding is estimated in the presence of 100 μM Dipyridamole. Membranes are filtered and washed, the filters are then counted to determine [^3^H]PK 11195 specifically bound. For reference, previously published results on rat TSPO showed binding affinities (K_i_) of DPA-713 and DPA-714 are 4.7±0.2 nM and 7.0±0.4 nM, respectively.[4]

# References

1. Banister SD, Wilkinson SM, Hanani R, Reynolds AJ, Hibbs DE, Kassiou M. A practical, multigram synthesis of the 2-(2-(4-alkoxyphenyl)-5,7-dimethylpyrazolo[1,5-a]pyrimidin-3-yl)acetamide (DPA) class of high affinity translocator protein (TSPO) ligands. Tetrahedron Letters. 2012;53:3780–3.

2. Cappelli A, Bini G, Valenti S, Giuliani G, Paolino M, Anzini M, et al. Synthesis and Structure–Activity Relationship Studies in Translocator Protein Ligands Based on a Pyrazolo[3,4- *b* ]quinoline Scaffold. J Med Chem. 2011;54:7165–75.

3. Klintworth R, de Koning CB, Michael JP. Practical Decagram-Scale Synthesis of a Lamellarin Analogue and Deprotection of Lamellarin Isopropyl Ethers. European Journal of Organic Chemistry. 2020;2020:3860–71.

4. Reynolds A, Hanani R, Hibbs D, Damont A, Pozzo ED, Selleri S, et al. Pyrazolo[1,5-a]pyrimidine acetamides: 4-Phenyl alkyl ether derivatives as potent ligands for the 18 kDa translocator protein (TSPO). Bioorganic and Medicinal Chemistry Letters. 2010;20:5799–802.
